# Health equity for persons with disabilities: a global scoping review on barriers and interventions in healthcare services

**DOI:** 10.1186/s12939-023-02035-w

**Published:** 2023-11-13

**Authors:** Mélanie Gréaux, Maria Francesca Moro, Kaloyan Kamenov, Amy M. Russell, Darryl Barrett, Alarcos Cieza

**Affiliations:** 1 Faculty of Education, University of Cambridge, Cambridge, UK; 2 Columbia University, New York, USA; 3 World Health Organization, Geneva, Switzerland; 4 University of Leeds, Leeds, UK

**Keywords:** Health equity, Disability, Persons with disabilities, Healthcare services, Access, Barriers, Interventions, Scoping review

## Abstract

**Background:**

Persons with disabilities experience health inequities in terms of increased mortality, morbidity, and limitations in functioning when compared to the rest of the population. Many of the poor health outcomes experienced by persons with disabilities cannot be explained by the underlying health condition or impairment, but are health inequities driven by unfair societal and health system factors. A synthesis of the global evidence is needed to identify the factors that hinder equitable access to healthcare services for persons with disabilities, and the interventions to remove these barriers and promote disability inclusion.

**Methods:**

We conducted a scoping review following the methodological framework proposed by Arksey and O’Malley, Int J Soc Res Methodol 8:19–32. We searched two scholarly databases, namely MEDLINE (Ovid) and Web of Science, the websites of Organizations of Persons with Disabilities and governments, and reviewed evidence shared during WHO-led consultations on the topic of health equity for persons with disabilities. We included articles published after 2011 with no restriction to geographical location, the type of underlying impairments or healthcare services. A charting form was developed and used to extract the relevant information for each included article.

**Results:**

Of 11,884 articles identified in the search, we included 182 articles in this review. The majority of sources originated from high-income countries. Barriers were identified worldwide across different levels of the health system (such as healthcare costs, untrained healthcare workforces, issues of inclusive and coordinated services delivery), and through wider contributing factors of health inequities that expand beyond the health system (such as societal stigma or health literacy). However, the interventions to promote equitable access to healthcare services for persons with disabilities were not readily mapped onto those needs, their sources of funding and projected sustainability were often unclear, and few offered targeted approaches to address issues faced by marginalized groups of persons with disabilities with intersectional identities.

**Conclusion:**

Persons with disabilities continue to face considerable barriers when accessing healthcare services, which negatively affects their chances of achieving their highest attainable standard of health. It is encouraging to note the increasing evidence on interventions targeting equitable access to healthcare services, but they remain too few and sparce to meet the populations’ needs. Profound systemic changes and action-oriented strategies are warranted to promote health equity for persons with disabilities, and advance global health priorities.

**Supplementary Information:**

The online version contains supplementary material available at 10.1186/s12939-023-02035-w.

## Background

The World Health Organization estimates that approximately 1.3 billion people today have significant disability [1]. Persons with disabilities experience worse health outcomes when compared to the rest of the population in terms of premature mortality, increased morbidity, and limitations in functioning [1–3]. For instance, persons with disabilities are more likely to die at a younger age than the rest of the population [4–7], have a higher incidence of communicable diseases and chronic health conditions [8–11], and limitations to functioning [12–14]. COVID-19 further highlighted these disparities in health outcomes: during the pandemic people with disabilities were more likely to get infected by COVID-19 and, when infected, had an increased risk of severe illness and mortality compared to persons without disabilities [15–17].

Many of the poor health outcomes experienced by persons with disabilities cannot be explained by the underlying health condition or impairment, but are health inequities driven by unfair societal and health system factors (1). The population-wide structural and social determinants of health that generate, sustain and even widen health inequities are well-documented [18–20], and disproportionately impact persons with disabilities (2). For instance, persons with disabilities are significantly more likely to live in poverty [21], under precarious housing conditions [22], and have lower levels of education [23] and employment [24] than their non-disabled counterparts. The accumulation of these mutually-reinforcing determinants of health not only amplifies the health and social disadvantage experienced by persons with disabilities, but is further compounded by additional factors that are uniquely associated with disability, such as ableism [25]. Importantly, the health inequities experienced by persons with disabilities are also associated with poor access to quality healthcare. For example, a study conducted in England and Wales demonstrated that avoidable deaths from causes amenable to good quality healthcare are more common in people with intellectual disabilities (37%) than in the general population (13%) [26].

In order to address health inequities, we need to better understand the underlying factors that lead to them, and particularly those related to access to healthcare services [3, 27, 28]. Many published reviews have already synthesized the barriers of access to healthcare services for persons with disabilities, highlighting a wide range of issues such as physical inaccessibility of health facilities [29, 30], unaffordable healthcare [31], untrained healthcare workforce [32, 33], and negative attitudes of healthcare workers [34]. However, these reviews are often restricted to specific healthcare services, countries or demographics. Importantly, no published review investigated these barriers alongside the interventions to remove them and promote disability inclusion in these services, which limits the identification of solutions to promote health equity for persons with disabilities worldwide.

The present scoping review is the first to provide a comprehensive global overview of access to healthcare services for persons with disabilities as characterized by both (1) the barriers that persons with disabilities face when accessing healthcare services, and (2) the interventions that have been implemented to remove these barriers and promote equitable access to healthcare for this population. By mapping this evidence according to different components of the health system [35] and other contributing factors to health inequities, we aim to provide insights to inform the actions that governments and other key stakeholders can take to advance health equity for persons with disabilities [19, 28], and respond more efficiently to the requirements of the United Nations Convention on the Rights of Persons with Disabilities (UNCRPD) [36].

## Methods

We conducted a scoping review to identify the existing evidence on the barriers that persons with disabilities face when accessing healthcare services and the interventions to remove them and promote disability inclusion. The scoping review approach was adopted for its use for ‘reconnaissance’ to explore a large, complex, and heterogenous topic [37]. We followed the scoping review methodological framework provided by Arksey & O’Malley [38], which stipulates five steps: (1) Identifying the research questions; (2) Identifying relevant studies; (3) Study selection; (4) Charting the data; (5) Collating, summarizing, and reporting the results. We conducted this review in accordance to the Preferred Reporting Items for Systematic Reviews and Meta-Analyses Extension for Scoping Reviews guidelines [39] (see Additional file 1) and the ‘Guidance for conducting systematic scoping reviews’ [37].

### Identifying the research questions

In this scoping review, we aimed to address two research questions: (1) What are the barriers of access to healthcare services experienced by persons with disabilities worldwide?, and (2) Which interventions have been implemented to remove these barriers and promote disability inclusion in healthcare services?

### Identifying relevant studies

#### Search strategy

Our search strategy was designed to investigate evidence from multiple and complementary sources. We searched two scientific databases, MEDLINE (Ovid) and Web of Science, which represent the highest scoring database combination accessible to the team after considerations of access and capacity constraints [40]. The search strategy was framed around the combination of three key concepts: (1) Accessibility, (2) Persons with disabilities; and (3) Healthcare services. We developed a list of key words for each concept using MeSH (Medical Subject Headings; National Library of Medicine) and other reviews on similar topics. We used Boolean, truncation, and proximity operators to construct and combine searches, and implemented adjustments as required to account for the specific functionalities of each database. The MEDLINE (Ovid) search strategy, which was run on October 13^th^, 2021, is provided in Additional file 2.

Additionally, we searched for evidence in websites of relevant organizations and governments using Google Search engine. We reviewed the websites of the two main global umbrella networks of Organizations of Persons with Disabilities and service providers, namely International Disability Alliance (IDA) [41] and International Disability and Development Consortium (IDDC) [42]. Since IDA and IDDC encompass a large number of local and regional organizations, we believe that this allowed us to get sufficient outreach. We also searched the Disability Evidence Portal [43]. Finally, we complemented these initial searches with a snowballing approach to review the websites of organizations mentioned in the webpages that we reviewed and included relevant information on the topic of health for persons with disabilities.

We also reviewed articles recommended by experts during consultations held by the World Health Organization for the development of the Global report on health equity for persons with disabilities [1]. Since all co-authors were involved in the planning of these consultations, and given their relevance to the objectives of the present scoping review, this provided us with a unique opportunity to identify further articles recommended by a range of stakeholders. These consultations were attended by over 1,250 experts worldwide, including persons with disabilities, representatives of Organizations for Persons with Disabilities, United Nations agencies, academics, services providers, and other experts. More details about these consultations are provided in Annex 2 of the Global report [1].

#### Selection criteria

We included publications from the last 11 years (2011–2022), with the meaningful threshold of 2011 being chosen due to the publication of the World Report on Disability [2] which attracted global attention to the inequities experienced by persons with disabilities. We included various publication types to capture the voices of different stakeholders and identify key themes and trends in the field of disability (qualitative, quantitative, and mixed-method peer-reviewed journal articles; systematic and scoping reviews; and reports produced by civil society organizations and governments). However, we excluded other publication types such as clinical guidelines, research protocols, commentaries, editorial comments, book chapters, conference abstracts and presentations. We included publications from any country and published in any language accessible to the research team (English, French, Italian, and Spanish). Publications were eligible if they documented the perceived or measured barriers of access to health services, and/or interventions addressing equitable access to healthcare services for persons with disabilities. We did not apply restrictions to types of underlying impairments or health services. The same selection criteria were applied for articles identified from the scientific databases, grey literature and consultations.

### Study selection

We run the search in the two scientific databases and deduplicated the articles. A single reviewer (MG) screened all titles and abstracts (*n* = 9,440). A second reviewer (MFM) screened a 10% random sample to determine inter-rater reliability (*n* = 944). A kappa score of *K* = 0.727 was reached, indicating substantial agreement [44]. Conflicts were resolved through discussion between three reviewers: MG, MFM, and KK. Then, the reviewers identified publications eligible for full-text review, categorizing them according to the main aim of the paper as being primarily relevant to RQ1 (barriers) or RQ2 (interventions). We used a 20% random sample of both sets of articles, weighed according to publication year, to ensure a balanced representation of studies addressing both research questions. This sampling strategy was adopted to better engage with a smaller selection of this large dataset, and completed according to considerations of content saturation. MG and MFM retrieved all articles included through this sampling strategy for full-text review. For the grey literature search, MG, MFM and AMR were assigned different sources, recorded each URL that was monitored on an Excel document with a mention ‘to be included’ or ‘to be excluded’ following assessment to the selection criteria. The additional articles suggested during the WHO-led consultations were recorded in another Excel document, and following the same selection process. The extraction of references and deduplication was conducted on the reference manager software EndNote 20.4, and screening was conducted on Rayyan [45].

### Charting the data

The research team developed a data charting form for this review. The following characteristics were extracted for each included article on barriers and interventions: author(s), year of publication, study contexts (defined by country, WHO region^1^ [46], and level of income), methodology, study population, intersectionality,^2^ type of healthcare service or intervention, and main outcomes. In cases where articles referred to both barriers and interventions, this was recorded as additional remarks and the data was extracted to inform both lines of enquiry. References were allocated a unique reference number.

### Collating, summarizing, and reporting the results

We synthesized the barriers and interventions of access to healthcare services for persons with disabilities according to the key components of the health system [35]: (1) health and care workforce; (2) health information systems; (3) health systems financing; (4) leadership and governance; (5) service delivery; and (6) essential medicines and equipment. We used this framework because it is widely adopted in global health strategic planning. Therefore, mapping our findings to this framework can support the identification and integration of solutions to advance disability inclusion in existing universal health coverage plans. Additionally, we considered the following components that are extrinsic to the health system but nonetheless critical to healthcare access: structural barriers, social determinants of health, and risk factors related to ill-health (such as tobacco and alcohol use, or physical inactivity). We did not undertake a quality appraisal of the included papers as this is not recommended practice for scoping review designs which aim to provide an overview of the field rather than assess its evidence [38].

## Results

### Overview

**Fig. 1:**
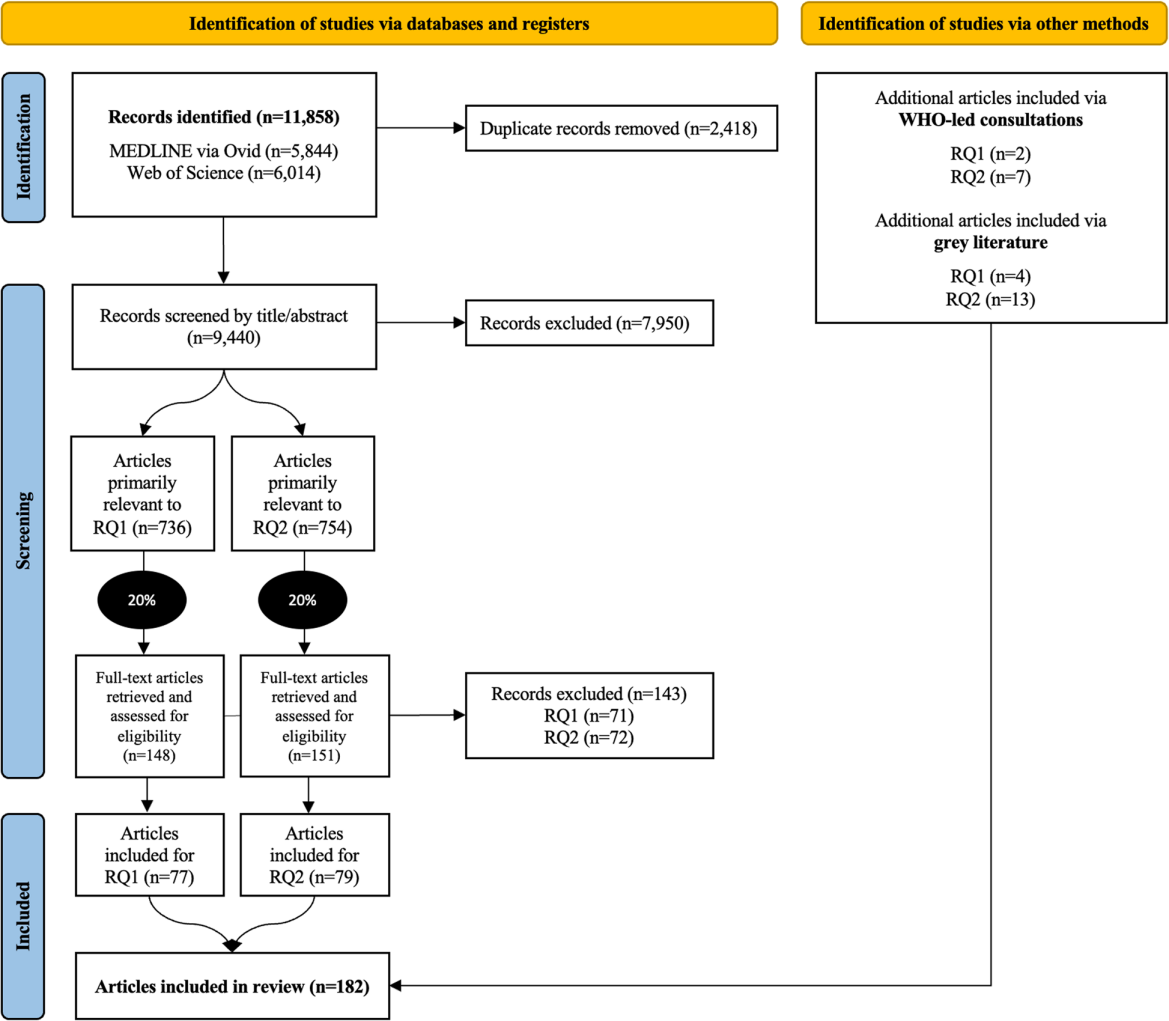
PRISMA flowchart indicating the study selection

Of 11,858 articles identified in the database search, we identified 736 and 754 articles at the title/abstract screening stage to primarily address RQ1 (barriers) and RQ2 (interventions), respectively. Following the 20% random sampling strategy in both sets of articles and full-text screening, we identified 77 and 79 articles to address RQ1 and RQ2 respectively. This indicated an inclusion rate of 52.0% and 52.3% of the samples retrieved after title-abstract screening. We included another six articles for RQ1 and 20 for RQ2 through consultations and grey literature search. In total, we included 182 articles in this review (Fig. 1). All articles were accessible in English, except for one paper written in French [49].

#### Barriers

**Fig. 2:**
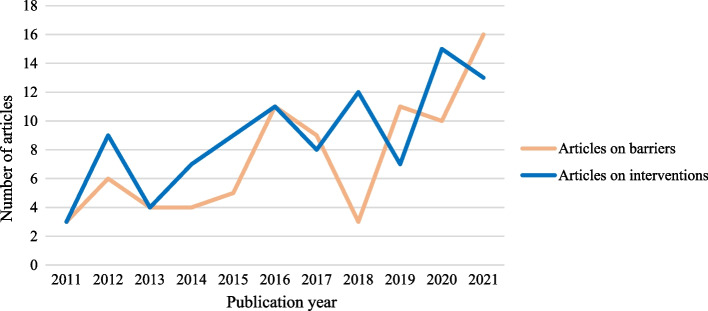
Frequency of articles on barriers and interventions based on publication year

The 83 included articles on the barriers of access comprised 77 articles from the database search and 6 from other sources. All 77 included articles from the database search were originally categorized for barriers (RQ1). From the articles categorized as primarily addressing RQ2 (interventions), most authors also mentioned the barriers of access to healthcare for persons with disabilities that they aimed to address (RQ1) but no novel barriers were identified that had not already been captured in the articles categorised as primarily addressing RQ1. These articles reported largely on the Americas (28.9%), European and Western Pacific regions (19.3% each), while the least represented region was Eastern Mediterranean (3.6%). Many articles were set in high-income countries (66.3%) and few in low-income countries (3.6%). About a third of the papers investigated access to general healthcare or primary care services (32.5%) while the rest focused on more targeted services. Qualitative research designs were used in half of these papers (50.6%), followed by mixed methods (25.3%), quantitative (15.7%), and review methods (8.4%). Most articles included persons with mixed types of disabilities (33.7%), followed by psychosocial disabilities (25.3%), physical (16.9%), intellectual (14.5%), and sensory disabilities (9.6%). Finally, almost half of the papers provided information about specific groups of persons with disabilities at risk of further marginalization (54.2%), with a stronger focus on women (19.3%) or children with disabilities (13.3%), but older persons (2.4%), migrants (1.2%) or persons with disabilities living in rural areas (3.6%) were less represented. Table 1 (in Additional file 3) details the characteristics of the included papers on barriers, and Fig. 2 shows the frequency of these articles based on publication year.

#### Interventions

The 99 included papers on the interventions comprised 79 articles from the database search and 20 from other sources. No new intervention was found in the sample of papers categorized as primarily addressing RQ1 (barriers). Similar to the articles on barriers, most articles were situated in the Americas (39.4%), Western Pacific (18.2%), and European regions (15.2%). The African (6.1%), South-East Asian (3.0%), and East-Mediterranean regions (1%) were scarcely represented. The articles reported largely on high-income countries (62.6%) and showed low representation of low-income countries (2.0%). Most articles focused on interventions for persons with psychosocial (61.6%) or mixed disabilities (20.2%), while interventions targeting persons with sensory (8.1%), intellectual (7.1%), or physical disabilities (3.0%) were few. Specific groups of persons with disabilities at risk of further marginalization were a consideration for 25.3% of papers and most particularly for children with disabilities (15.2%), but less so for women with disabilities (6.1%), those with minoritized ethnic backgrounds (2.0%), older persons or persons with disabilities living in rural areas (1.0% each). Most intervention types related to training and education (38.8%), and care coordination (28.3%). Table 2 (in Additional file 3) details the characteristics of the included papers on interventions, and Fig. 2 shows the frequency of these articles based on publication year.

## Health system-related barriers and interventions

### Health and care workforce

The evidence on healthcare workforce-related barriers was evenly distributed across all types of disability, and particularly well-documented for women (22.7%) and children with disabilities (18.2%) in rehabilitation services (22.7%) and maternal care (15·2%). The majority of included papers (79.5%) exposed the limited human resources, lack of training and skills, and negative attitudes of the health and care workforce that hindered access to healthcare services for persons with disabilities. Many articles showed that limited staff [50–59] and high staff turnover [60–62] were widespread problems that disadvantaged persons with disabilities who often needed longer or more frequent sessions so that their health needs could be met [54, 59]. Even when available, healthcare workers often lacked the knowledge, skills, experience, and confidence to care for persons with disabilities, including on providing reasonable accommodations [31, 49, 50, 52, 54–56, 58–96]. A large number of articles referred to issues of discriminatory attitudes and behaviours by healthcare workers towards persons with disabilities across all healthcare services, regions, and levels of income [31, 51, 53, 55, 57, 58, 61–63, 65, 66, 68, 70–74, 76–79, 81–84, 87, 88, 94, 95, 97–106]. This was illustrated by healthcare workers’ refusal to provide care for persons with disabilities or to adopt reasonable accommodations [31, 98, 106], negative assumptions around the capacity of persons with disabilities to engage in their own care [62, 70, 71, 73, 76, 94, 104, 105], or disrespect for persons with disabilities’ wishes of care [80]. Additionally, the communication challenges between healthcare workers and persons with disabilities represented critical barriers to equitable healthcare [31, 49–51, 55, 59, 68, 69, 71, 73, 74, 78, 82–85, 87, 90–93, 97, 99–102, 104, 107–109], which particularly affected persons with cognitive [85], developmental [69], or communication disabilities [51, 59].

All 19 included interventions targeting the healthcare workforce focused on the training and education of healthcare workers. These interventions largely focused on building the capacity of the healthcare workforce to care for persons with psychosocial disabilities (63.2%) and children with disabilities (15.8%), but only three were scaled up nationally (15.8%). Education programmes aimed to improve healthcare professionals’ knowledge on disability issues [110–117], inclusive communication skills [118], or to address negative attitudes [110, 111, 119–123]. Most interventions increased healthcare professionals’ knowledge and skills, but rigorous assessment of their application and impact in practice was often missing. Furthermore, most of these interventions were focused on the care for persons with psychosocial disabilities, which indicated gaps in healthcare workforce training for other groups of persons with disabilities with different needs. Importantly, only a few interventions incorporated a human rights-based approach to the care of persons with disabilities [111, 124] and the involvement of persons with disabilities, in line with the UNCRPD [117].

### Health information systems

Few articles shed light on the barriers and interventions around the health information systems (33.7%), and largely focused on issues of care coordination. The lack of reliable disability data in healthcare services [66] and the lack of a system to record the reasonable adjustments to care for persons with disabilities [67] were reported. Care coordination was often limited due to poor referral systems [49, 52, 68] and inefficient exchange of information between healthcare providers, which would cause delays in the provision of care [61, 68, 69, 125, 126]. Most evidence documented how this issue affected children with disabilities (25.0%) and persons with psychosocial disabilities (28.6%) in general healthcare services (53.6%), but the impact on other groups and services remained largely unknown.

Only two interventions were identified on health information systems, and only one was scaled up at the regional level. Both were set in high-income countries (USA [127] and Canada [128]) and highlighted the potential of digital solutions, such as electronic patient records, to improve healthcare for persons with psychosocial or mixed disabilities.

### Health systems financing

Many included articles covered the financial barriers of healthcare access for persons with disabilities (61.4%), with these barriers being disproportionately reported in low- and middle-income countries. While less than half of the articles from high income countries reported these financial issues (48.3%), almost all articles from low- and middle-income countries did (92%). The evidence focused particularly on how these barriers affected persons with psychosocial disabilities (31.4%), women (19.6%) and children with disabilities (17.6%) as they accessed general healthcare services (31.4%) and rehabilitation services (25.5%). The healthcare costs [31, 49, 50, 52, 56, 58, 60, 61, 63, 64, 76, 83, 86, 89, 91, 95, 97–100, 102, 103, 129–138], the lack or limited coverage by health insurance [31, 57, 58, 72, 82, 88, 105, 139, 140], and out-of-pocket payments [56, 57, 72, 78, 82, 131, 134, 140] often prevented persons with disabilities from accessing timely care [98, 134, 135, 140]. For example, persons with disabilities are from 4·5 to 7·2 times more likely to have unmet need for mental health due to cost [138]. There was a global lack of investment in resources to promote accessibility for persons with disabilities in health systems [49, 52, 53, 57–59, 62, 69, 81, 83, 86, 89, 126], such as the lack of investments in accessible equipment [52] or discriminatory decisions surrounding health insurance schemes [81]. Furthermore, persons with disabilities often faced difficulties with the administrative requirements and processes needed to obtain insurance or access disability funding [59, 101, 103, 141].

A total of seven interventions with a primary focus on inclusive health systems financing were identified, including six scaled up at the national level. All were developed in high-income countries from the Americas (USA), European (Germany) and Western Pacific regions (Australia). These interventions aimed to improve health insurance packages, mostly for persons with psychosocial disabilities (57.1%) [142–145], children with disabilities (28.6%) [146, 147], and all persons with disabilities (28.6%) [148]. However, the evidence on their impact to healthcare access was mixed. Furthermore, targeting specific groups known to be at higher risk of financial hardship, such as for women and older persons with disabilities, or persons with intellectual disabilities, was missing.

### Leadership and governance

The barriers associated with this component of the health system received the least evidence globally, with 22.9% articles referring to issues of leadership and governance from countries in various regions and with different income levels. The reported lack of governmental or managerial leadership on disability inclusion highlighted prioritization issues among decision-makers in the health sector [52, 63, 83, 86]. The lack of alignment of policies between services [126] and limited awareness of healthcare workers on such policies and guidelines [53, 58, 68, 83, 89, 92, 101] emphasized implementation issues. The lack of disability guidelines and legislation enforcement [65, 66, 72, 86] and accountability mechanisms for decision-makers in the health sector [67] often led to dependence on other sources of support, such as provided by small NGOs and Organizations of Persons with Disabilities.

Among the eight interventions identified to improve issues of leadership and governance, most related to the implementation and evaluation of disability-inclusive health policies and guidelines in the health sector nationally. Interventions from Israel [149], South Africa [150], Ukraine [151] and the USA [143, 147, 148, 152] were identified, but their impact was difficult to appraise. Most of these interventions did not distinguish persons with disabilities on the basis of other individual factors (62.5%) nor the specific needs of different groups of persons with disabilities (62.5%).

### Service delivery

Barriers in service delivery were identified in all types of services, with 89.2% of the included articles reporting on issues of service availability and quality. Groups of persons with disabilities at risk of further marginalization were investigated in over half of these papers, with a strong focus on the barriers experienced by women with disabilities (23.0%). Most of the articles documented the barriers found in general healthcare (29.7%), rehabilitation (21.6%), and mental healthcare services (20.3%) for people with mixed (32.4%) and psychosocial disabilities (28.4%). The lack or limited availability of healthcare services [31, 50, 52, 53, 55–59, 63, 72, 94, 97, 98, 101, 102, 106, 126, 129, 131, 137, 138, 140, 141, 153, 154], especially in rural areas [52, 56, 101], often segregated the most marginalized persons with disabilities [31, 50, 56, 63, 72, 98, 101, 131, 138, 154]. The lack of care coordination [57, 59, 61, 78, 79, 86, 103, 125, 126] and service fragmentation [57, 64, 71], particularly as it related to mental and physical care [49, 86, 92] and multisectoral collaboration [69, 73, 86, 92, 129], was evident on the global scene and further disadvantaged persons with disabilities who may present with more complex health needs.

The lack of accessible architectural designs often impeded access for persons with disabilities [55, 61, 63, 66, 70, 72, 76, 77, 82, 88, 95, 100, 102, 107, 125], particularly those with mobility difficulties. This was illustrated by the lack of accessible consultation rooms [61, 72], toilets or washing rooms [55, 61, 63, 70, 95, 102], ramps [95, 102], routes and parking areas [63, 72]. The sensory distractions in busy clinical environments [57, 73] could be particularly challenging for persons with communication [51] and psychosocial difficulties [57, 73]. The time constraints regulating healthcare provision, such as inflexibility of appointment times [61, 76, 82, 92, 155], long waiting to secure medical appointments [50, 69, 129], or short consultation times [49, 107, 125], hindered the opportunities to coordinate or provide timely care with needed accommodations for persons with disabilities [69, 88]. The limited or inconsistent information about health services [53, 56, 57, 63, 71, 72, 76, 78, 80, 89, 93, 94, 101, 103, 125, 134], and inaccessible medium of communication could widen this gap [72, 76, 80]. This was particularly evident in the difficulties to access any or good-quality sign language interpreters [55, 63, 72, 87, 88, 106, 109] and augmentative and alternative communication tools [51]; and negatively impacted the care of persons with sensory [72, 87, 106], cognitive [60], or communication difficulties [51].

A total of 53 interventions targeting disability-inclusive health service delivery were included, including 24.5% scaled up nationally. Most interventions aimed to promote the coordination of health service provision for persons with psychosocial disabilities (67.9%) and children with disabilities (15.1%) [156–160]. Programmes in the USA supporting the integration of physical and mental healthcare [143, 145, 157, 161–163] were evaluated, but their effects on healthcare utilization, costs and outcomes were mixed [164]. In the UK, attempts to embed routine health checks for persons with disabilities in primary care showed conflicting results [158, 165]. Beyond the health sector, multisectoral coordination was also considered, such as with non-government organizations [166], traditional and faith-based healers [167–169], or the police [170]. The evidence demonstrated that task-sharing approaches [166, 171, 172], mobile service delivery in the community [173], and telehealth [121, 128, 174, 175] could represent promising solutions to improve healthcare service delivery for persons with disabilities.

### Essential medicines and equipment

The lack of accessible or specialized medical and rehabilitation equipment, products and devices in healthcare services were frequently mentioned as impactful barriers for persons with disabilities [52, 54, 58, 59, 61–63, 66, 70, 72, 76, 77, 81, 88, 94, 95, 98, 102, 129, 131, 140]. This was illustrated by the lack of adjustable examination tables [66, 70, 72, 76, 88, 94, 95, 140] or chairs [61], lifts or transfer devices [72, 140], weight scales [66, 140], delivery beds [70, 102], mobility aids [70, 102], and out-of-stock medication [63]. Even when available, this equipment could be misused [66]. These barriers were reported in countries across all world regions and with different levels of income, and particularly in rehabilitation services and general healthcare for persons with physical disabilities (30.3%).

No interventions were identified to facilitate access to medicines and accessible equipment in healthcare services for persons with disabilities, which underscores an urgent systemic gap.

## Other factors

### Structural factors

Beyond health systems, the impact of structural factors on access to healthcare services for persons with disabilities was evident. The negative attitudes towards persons with disabilities across all strata of society fuelled inequitable access to healthcare [55, 57, 59, 64, 79, 80, 82–84, 95, 98, 102, 106, 125, 130, 131, 134, 137]. Socio-cultural discriminatory beliefs about disability [59, 64, 83, 90, 102, 129, 134, 135, 153] often influenced help-seeking behaviours [64, 108, 129, 135, 137]. Family members of persons with disabilities may perpetuate these beliefs and attitudes [50, 55, 64, 68, 80, 83, 84, 95, 98, 102, 108, 129, 134, 135, 137] and hide their relatives with disabilities [98, 135], which could cause significant delays in accessing services or even prevent care [79, 98, 108, 134, 135]. Internalized stigma by persons with disabilities could also impact their access to healthcare services [31, 60, 83, 95, 105, 106, 108, 132, 133, 137, 176, 177]. This was particularly well evidenced in articles focusing on access to mental health services [83, 106, 108, 132, 133, 177] and sexual and reproductive health services [95, 176].

In total, 18 interventions targeting the negative societal attitudes towards persons with disabilities were identified [178–182], including 72.2% focusing specifically on persons with psychosocial disabilities and only 16.7% being scaled up at the national level. A few public campaigns targeting stigma to promote the use of mental health services were scaled up nationally in the USA [183] and UK [184]. On a global scale, the WHO QualityRights initiative aimed to improve societal attitudes towards persons with psychosocial disabilities and promote access to quality mental healthcare through a human right-based approach, with positive results [124].

### Social determinants of health

The social determinants of health played a critical role in inequitable access of healthcare services for persons with disabilities. Most notably, poverty experienced by persons with disabilities [57, 86], often exacerbated by unemployment [56, 95, 131] or homelessness [73], could limit care-seeking behaviours. This was particularly relevant for persons with psychosocial disabilities. Persons with disabilities were often dependent on the benevolence of their caregivers to access health services, which resulted in issues of autonomy and confidentiality that were well evidenced for children, girls and women with disabilities [81, 99, 130, 137]. Additionally, persons with disabilities and their caregivers rarely benefited from educational opportunities to develop their health literacy [31, 52, 53, 57, 59, 62, 64, 73, 74, 76, 79, 80, 83, 87, 106, 108, 129, 134, 137], hence lacked the knowledge to identify their health needs [31, 177] and services that can help [31, 66, 82, 131, 177].

Persons with disabilities frequently faced issues related to the lack or limited availability of accessible transportation to reach healthcare services [31, 50, 54, 60, 76, 82, 89, 98, 101, 102, 138–140, 176, 185, 186], which could be further prohibited by transportation costs [31, 56, 60, 81, 89, 98, 101, 102, 131, 134, 135, 186], the lack of accommodations for physical [81] or psychosocial needs [62], and the risk of violence [186] in public services.

Importantly, intersectional issues leading to inequitable access of healthcare services for persons with disabilities were reported. Those living in rural areas [52, 86] were further disadvantaged due to the lack of community healthcare, which also resulted in additional travel costs [56]. Women and girls with disabilities faced additional challenges, such as dependence on male family members around health-seeking decisions which could be highly problematic in the context of domestic abuse [80] and access to sexual and reproductive healthcare services. Specific barriers faced by persons with disabilities from minority ethnic groups [57, 60, 91] were highlighted, including language barriers [91, 92], the lack of culturally appropriate information or dependence on personal networks to arrange care [60]. Lastly, immigrants and refugees with disabilities [90, 108, 153] experienced additional issues of trust with services in a ‘foreign system’ [90, 153], the fear of the consequences on their right to stay in the country if they access services [90], and language and cultural barriers [90, 108, 153].

Among the 29 interventions identified to improve access of healthcare services for persons with disabilities through the social determinants of health, almost half of these interventions focused on persons with psychosocial disabilities, and only 20.7% were scaled up nationally. The majority targeted the education of caregivers [156, 187–189] and persons with disabilities [187, 189–194], mostly addressing issues of stigma [178, 195], self-stigma [178, 196–199], social support [200, 201], and developing the skills and knowledge of persons with disabilities [187, 189–194]. Other education programmes targeted the health advocacy and empowerment of persons with disabilities [190, 191, 193, 202] with a component of peer-support [203]. For example, the W-DARE project in the Philippines aimed to increase access to sexual and reproductive health information and services for women with disabilities through participatory research [193, 202]. A few interventions supported the development of accessible health information disseminated outside of healthcare services [187, 204–208] and mostly included the active involvement from persons with disabilities, but there was a considerable discrepancy between the large need and the limited reach of these small-scale interventions. Additionally, a few interventions adopted a multisectoral approach to target the social determinants of health for persons with disabilities. One such example is the Housing First approach implemented in North America to support persons with psychosocial disabilities [209]. There were interventions aimed at addressing transportation barriers for persons with disabilities, such as the Journey Access Tool in Cambodia [210] and the Transport*MY*patient programme in Tanzania [211]. Lastly, the development of telehealth services was highlighted as a promising solution for persons with disabilities living in rural areas [101, 131].

### Risk factors

Persons with disabilities had heightened health risks such as medical complications or comorbid conditions [83, 85, 109], which could directly impact their access to healthcare services. For example, they may be feeling too unwell or sick to arrange care and access services [85, 132, 177], or prioritizing certain health needs over others [31, 60, 89, 105, 129]. Additionally, the overshadowing of symptoms by healthcare workers could lead to inefficient, even harmful, services for persons with disabilities in healthcare settings [89, 95]. The majority of this evidence explored the risk factors experienced by persons with psychosocial disabilities, but little was reported on the needs of other groups of persons with disabilities at risk of further marginalization. No interventions targeting risk factors were identified to improve access to healthcare services for persons with disabilities.

## Discussion

This scoping review is the first of its kind to present a global overview of the barriers that persons with disabilities face when accessing healthcare services, as well as the interventions that are implemented to remove them and promote inclusive services. It yields critical insights for the global public health community on what is needed to improve healthcare access for the 1.3 billion people with disabilities worldwide [1], in order to advance health equity and catalyze efforts to achieve global health targets. Four key trends are highlighted below, and their implications for relevant stakeholders discussed.

Firstly, this review highlights a positive trend of increased publication rates of articles on the barriers of access to healthcare services over the past decade, which illustrates a heightened interest in factors leading to health inequities for persons with disabilities. A similar trend is observed for the articles on interventions, however these do not readily map onto the actual needs of persons with disabilities. For example, our results show that the common barriers faced by persons with disabilities are often related to issues of transportation to access healthcare services [31, 50, 60], widespread difficulties around communication [49, 51, 55], and the lack of accessible equipment in healthcare facilities [52, 70, 76]. These findings are echoed in previous reviews on the topic [212, 213], but our review also demonstrates that these barriers remain largely unaddressed by the interventions to promote disability inclusion. Additionally, interventions are often siloed small-scale projects with little indication on sustainability, scaling up or cross-sectoral engagement. The lack of investment and evaluation of larger-scale interventions comes in stark contrast with the high and increasing prevalence of persons with disabilities globally [1]. Therefore, we encourage service providers and policymakers to invest in and align their efforts in global, national and local health planning to meet the needs of persons with disabilities. Interventions that are expected to have the most impact are those that address the most pressing needs reported by persons with disabilities, strategize solutions across all components of the health system to meet these needs, and strengthen collaboration with multisectorial stakeholders to tackle the complex issues of health inequities beyond the health sector [214].

Secondly, it is encouraging to note the focus on certain groups of persons with disabilities at a higher risk of discrimination and marginalization in about half of the articles included on barriers. However, this trend is not replicated in the design of interventions to promote inclusive healthcare services. This means that the solutions that are being implemented may not reach some of the most marginalized groups of persons with disabilities, such as older persons with disabilities [215], ethnic minorities [216], women and girls or gender diverse persons [217], children [23], refugees and immigrants with disabilities [218]. While our findings provide only limited insights on how intersectionality interplays with health equity, it is essential to recognize that the health needs and priorities of different groups of persons with disabilities can differ widely and require tailored actions. For example, women with disabilities face unique challenges in sexual and reproductive healthcare services, such as the lack of accessible delivery beds [70], stigma towards their sexuality or even forced sterilization [102, 219]. Importantly, addressing the barriers faced by the most marginalized groups of persons with disabilities can foster health equity for everyone. This is because the same barriers often hinder access to healthcare services for other marginalized groups, such as older adults, people with noncommunicable diseases, or refugees [1]. We recommend service providers, policymakers, and stakeholders consult with persons with disabilities with a wide range of intersectional identities in order to better understand and address their unique health needs and intersectional mediating and risk factors to improve access to healthcare services. Special considerations should be given to the needs of women and girls, sexual and gender minority groups, children and older persons, ethnic minorities, and immigrants and refugees with disabilities to accelerate the prioritization and implementation of the most impactful strategies, and optimize resource allocation towards health equity [1]. For example, Ageing- and Disability-friendly cities are promising initiatives to identify and address the needs of older citizens with and without disabilities to inform more inclusive urban spaces, promote participation and healthy living in communities [220].

Thirdly, the lack of involvement of persons with disabilities and their representative organizations is observed in most interventions, and may well contribute to widening gaps and needs remaining unmet [193, 202]. In this review, we incorporated information that was shared during consultations organized by the World Health Organizations as part of the development process of the Global report on health equity for persons with disabilities [1]. While our results cannot claim to reflect the views and knowledge of those who participated in these consultations, this additional step was important to operationalize the principles of the UNCRPD towards more engagement with persons with disabilities and their representative organizations. In fact, these consultations, led to the increased representation of interventions promoting human right-based and consultative approaches in our results [124, 168, 191]. The meaningful engagement of persons with disabilities and their representative organizations in every effort to promote inclusive access to healthcare – and especially in leadership roles – is essential to progress this agenda [1]. Promoting innovative and empowering participatory processes can catalyze efforts to address health equity for persons with disabilities more meaningfully and efficiently [1, 221]. For example, the W-DARE programme on improving the sexual and reproductive health of women with disabilities in the Philippines successfully demonstrates the benefits of inclusion and participatory methods on care quality, impact, team capacity and commitment [193, 222]. We advise researchers, policymakers and services providers to strengthen collaboration with persons with disabilities and their representative organizations to accelerate the identification, prioritization and implementation of strategies, and promote innovation and capacity-building within and beyond the health sector [202]. We encourage governments and decision-makers in the health sector to set expectations and establish a collaboration mechanism to work efficiently with Organizations of Persons with Disabilities. The Government of Australia, through its digital transformation strategy, sets a good example by stating the inclusion of persons with disabilities and other groups in drafting policies and in the digital design process [223].

Fourthly, our review further emphasizes the uneven representation of countries in the research evidence on disability and healthcare provision. The evidence is largely skewed towards high-income countries, which comprises about two thirds of the articles included on barriers (66.3%) and interventions (62.6%). This represents a clear limitation since an estimated 80% of persons with disabilities worldwide live in low- and middle-income countries [1], and because interventions are highly sensitive to resources. Furthermore, this trend is compounded by the fact that intervention impact is inconsistently evaluated or done according to differing, incomparable criteria to measure success. For example, the profile of persons with disabilities who are eligible to benefit from the intervention may be mentioned, but it is often unclear whether the interventions reached people and improved their health and wellbeing [149]. Together, these gaps are detrimental to the identification of appropriate, wide-reaching, and impactful interventions to address health equity in least advantaged settings where the need is the highest. They expose the need to carefully consider and optimize the distribution of resources to advance health equity for persons with disabilities globally, and to put in place a systematic and transparent evaluation mechanism to assess impact. To address this, global health decision-makers and funders, in close collaboration with Organizations of Persons with Disabilities, have a key role to play in overseeing and coordinating the distribution of resources, building the capacity of country partners, prioritizing the most disadvantaged, and monitoring progress on health equity for persons with disabilities worldwide. Existing networks, such as the Global Action on Disability Network [224], that bring multiple stakeholders together can facilitate this coordination towards inclusive international development.

By uncovering these global trends around access to healthcare services for persons with disabilities in this review, we also expose the need to develop a comprehensive global research agenda to better mobilize, guide, and advance actions for health equity for persons with disabilities. The recently published WHO Global report on health equity for persons with disabilities already provides evidence-based recommendations for governments, researchers, and other key stakeholders to promote disability inclusion in the health sector [1], such as through data disaggregation [225], or the meaningful participation of persons with disabilities at all stages of the research cycle and in decision-making roles [221]. To complement these recommendations, a global research agenda is needed to articulate a clear vision and address the existing gaps in a sustainable manner.

To maximize impact, this research agenda should prioritize the generation of evidence that will most readily promote synergies with existing national and global health systems planning, and stimulate health policy and systems research impact [226]. In addition, it should concentrate on the potential of disability inclusive strategies to positively impact access to quality healthcare services for the wider population and other marginalized groups. Importantly, the development of this agenda requires the close collaboration and engagement of multisectorial partners and research networks to better address the deep and multidimensional roots of health inequities, with persons with disabilities and their representative organizations at the forefront of such endeavor [36]. Last but not least, it is essential for this research agenda to stimulate a wide ambition for health equity: one which goes beyond the scope of health systems to inspire and capture change at the societal level. For example, generating evidence on national budgeting priorities and mechanisms can allow to advocate for inclusive investments in the health sector and beyond more effectively. Creating interdisciplinary research networks can promote cross-sectoral coordination to foster impact to the health and well-being of persons with disabilities (e.g., promoting the implementation of inclusive public health interventions in schools to reach children with disabilities).

In conclusion, it needs to be mentioned that this scoping review was conducted under certain limitations. First, while the research team carefully selected two of the most prolific and relevant scientific databases in the field, it would have been desirable to run the search in more databases but this was not possible due to access and capacity constraints. Second, we used a random sample of the evidence to better engage with a selected set of articles and reflect on global trends in this field, which means that other relevant articles may have been missed. We reached a saturation point, as determined by the identification of redundant patterns in the information extracted from the included articles and through discussions with all co-authors on these trends. As such, the findings should provide an overview of the global evidence and fulfil the purpose of a scoping review approach, but more targeted lines of enquiry are warranted to complement these findings. Third, we included articles in only four languages, which may have limited the representation of evidence from certain countries. Fourth, we recognize that many of the interventions implemented to promote access to healthcare services for persons with disabilities may not have reached the scientific or grey literature. Lastly, the lens of the health system components was useful to present evidence on this topic and derive recommendations for leaders and decision-makers in the health sector. However, this framework may not capture all the perspectives, nuances and mechanisms that regulate health equity for persons with disabilities, especially as they related to the complexities of factors informing health-seeking behaviours. These aspects should be considered in further research to complement the findings of our review, and in the development of conceptual frameworks on health equity. Finally, future studies will benefit from the close collaboration with grassroot non-governmental and civil society organizations to inform contextualised situation analyses, identify unpublished initiatives, and complement the findings of this review. The influential role of these partners has been particularly well demonstrated in the responses to health emergencies and COVID-19 [15, 227], thus revealing their critical function to achieve health equity for persons with disabilities.

## Conclusion

Persons with disabilities continue to face considerable barriers when accessing healthcare services, which negatively affects their chances of achieving their highest attainable standard of health [36]. It is encouraging to note the increasing evidence on interventions targeting equitable access to healthcare services, including some demonstrating strong alignment with the UNCRPD. However, they remain too few and sparse to meet the needs of over 1.3 billion people with disabilities globally [1]. This evidence calls for a radical change in the way that disability inclusion is considered, integrated, and sustained in health system planning. Beyond access to healthcare services, our findings indicate that wider structural change on how the health system and society can address health equity for persons with disabilities is needed to tackle current and anticipated global health priorities. Investigating intersectional mediating and risk factors is critical to reach a more nuanced understanding of the mechanisms that regulate the health inequities experienced by the most marginalized groups of persons with disabilities. Collaboration should be facilitated to drive innovation and impact: researchers, decision-makers in the health sector, Organizations of Persons with Disabilities, bilateral donors and other key stakeholders all have a role to play to drive the agenda of health equity for persons with disabilities.

## Supplementary Information

**Additional file 1.** PRISMA Extended checklist for scoping reviews.

**Additional file 2.** MEDLINE (Ovid) search strategy.

**Additional file 3.** Characteristics of the included studies.

## Data Availability

All relevant data are mentioned within the manuscript and supplementary materials, and the full references of the included papers in this review are provided.

## Footnotes

1 The six WHO regions are (1) African Region; (2) Region of the Americas; (3) South-East Asia Region; (4) European Region; (5) Eastern Mediterranean Region; (6) Western Pacific Region.

2 We define intersectionality when individuals are socially situated through multiple overlapping identities that intersect with systems of oppression and marginalization (e.g., gender, race, social class, disability), which can lead to unique experiences of health and an increased risk of health disparities. With this definition, we acknowledge the mechanisms of oppression and marginalization that contribute to health inequities for persons with disabilities, and move away from reductionist and stigmatized views of persons with disabilities. To extract information on intersectionality, we consulted the UN list of vulnerable groups (e.g., women, LGBTQI + , race, or migrants) and complemented it with the multiple identities of persons with disabilities elaborated in the UNCRPD (e.g., children with disabilities). [47] Kelly C, Dansereau L, Sebring J, Aubrecht K, FitzGerald M, Lee Y, et al. Intersectionality, health equity, and EDI: What's the difference for health researchers? International Journal for Equity in Health. 2022;21(1). [48] United Nations. Vulnerable groups: who are they? 2023 [3 October 2023]. Available from: https://www.un.org/en/fight-racism/vulnerable-groups. [36] United Nations. Convention on the Rights of Persons with Disabilities (UNCRPD). New York; 2006.
