# Supplementary material for: Health equity for persons with disabilities: a global scoping review on barriers and interventions in healthcare services
Source: Int J Equity Health. 2023 Nov 13;22:236. doi: 10.1186/s12939-023-02035-w (PMC10644565; doi:10.1186/s12939-023-02035-w)
Supplement: Supplementary file 2 — Additional file 2. MEDLINE (Ovid) search strategy. [file 12939_2023_2035_MOESM2_ESM.docx]

**Additional file 2:** MEDLINE (Ovid) search strategy

Ovid MEDLINE(R) and Epub Ahead of Print, In-Process, In-Data-Review & Other Non-Indexed Citations, Daily and Versions(R) <1946 to October 12, 2021>

| **Accessibility** | 1 | exp Health Services Accessibility/ | 119136 |
| --- | --- | --- | --- |
|  | 2 | exp Healthcare Disparities/ | 19920 |
|  | 3 | (Health Services Accessibility or Healthcare Disparities or Access* or Barrier* or Challenge* or Equal* or Equit* or Exclusi* or Imped* or Inaccess* or Inclusi* or Inequal* or Inequit* or Inhibit* or Unequit* or Uptak* or Utilis* or Utiliz* or Obstacle*).ti,ab. | 5683645 |
| **Persons with disability** | 4 | exp Disabled Persons/ | 69140 |
|  | 5 | Intellectual Disability/ | 56829 |
|  | 6 | Developmental Disabilities/ | 21318 |
|  | 7 | Amputees/ | 3856 |
|  | 8 | Disabled Children/ | 6666 |
|  | 9 | Persons with Mental Disabilities/ | 3671 |
|  | 10 | Mentally Ill Persons/ | 6344 |
|  | 11 | Persons With Hearing Impairments/ | 2911 |
|  | 12 | Visually Impaired Persons/ | 2606 |
|  | 13 | Learning Disabilities/ | 14402 |
|  | 14 | Hearing Loss/ | 17777 |
|  | 15 | Deafness/ | 27201 |
|  | 16 | Blindness/ | 20461 |
|  | 17 | (Disabled Persons or Intellectual Disability or Developmental Disabilities or Amputees or Disabled Children or Persons with Mental Disabilities or Mentally Ill Persons or Persons with Hearing Impairments or Visually Impaired Persons or Learning Disabilities or Hearing Loss or Deafness or Blindness).ti,ab. | 128858 |
|  | 18 | ((Acoustic or Cognitive* or Communication or Developmental* or Ear* or Eye* or Hearing or Intellectual* or Invisible or Language or Learning or Mental* or Mental Health or Mobil* or Motor or Neurodevelopmental* or Neuro-developmental or Neurological* or Physical* or Psychiatric or Psychological* or Psychosocial* or Sensory or Speech or Vision or Visual*) adj1 (Deficienc* or Delay* or Difficult* or Disabilit* or Disabled or Handicap* or Ill* or Impair* or Loss or Retard*)).ti,ab. | 395327 |
|  | 19 | ((Adolescent* or Adult* or Child* or Men or People or Person* or Teen* or Women) adj3 (Disabilit* or Disabled or Handicap* or Impair* or Deaf or Blind)).ti,ab. | 73730 |
| **Healthcare services** | 20 | Health Services for Persons with Disabilities/ | 143 |
|  | 21 | Health Services/ | 25982 |
|  | 22 | Primary Health Care/ | 84484 |
|  | 23 | Community Health Services/ | 32518 |
|  | 24 | Reproductive Health Services/ | 2181 |
|  | 25 | Family Planning Services/ | 25507 |
|  | 26 | Maternal Health Services/ | 14936 |
|  | 27 | Child Health Services/ | 21092 |
|  | 28 | Adolescent Health Services/ | 5776 |
|  | 29 | Mental Health Services/ | 36185 |
|  | 30 | Psychiatric Rehabilitation/ | 542 |
|  | 31 | Rehabilitation/ | 18587 |
|  | 32 | Occupational Health Services/ | 10690 |
|  | 33 | Speech Therapy/ | 6588 |
|  | 34 | Language Therapy/ | 1976 |
|  | 35 | Cancer Care Facilities/ | 5740 |
|  | 36 | Cardiac Rehabilitation/ | 2965 |
|  | 37 | Neurological Rehabilitation/ | 1213 |
|  | 38 | Stroke Rehabilitation/ | 15087 |
|  | 39 | ((Community or Primary or General or Sexual or Reproductive or (Sexual and reproductive) or SRH or Contracept* or Antenatal or Prenatal or Postnatal or Maternity or Family planning or Gynaecolo* or Gynecolo* or Obstetric or Pregnancy or Midwifery or Maternal or New-born or Infant* or Bab* or Child* or Adolescent or Paediatric or Paediatric or Women or Mother* or Family or Mental or Psychiatric or Psychiatry or Psychological or Psychopathology or Psychotherapy or Rehabilitation or Physiotherapy or Physical Therapy or Occupational Therapy or Speech Therapy or Language Therapy or Noncommunicable Disease* or Non Communicable Disease* or Non-Communicable Disease* or Diabetes or Cancer or Oncolog* or Chemotherapy or Radiotherapy or Cardiac or Cardiovascular or Cardiolog* or Neurological or Stroke* or Respiratory) adj1 (Health service* or Health care or Healthcare or Care or Care service* or Service*)).ti,ab. | 265127 |
|  | 40 | 1 or 2 or 3 | 5753292 |
|  | 41 | 4 or 5 or 6 or 7 or 8 or 9 or 10 or 11 or 12 or 13 or 14 or 15 or 16 or 17 or 18 or 19 | 575865 |
|  | 42 | 20 or 21 or 22 or 23 or 24 or 25 or 26 or 27 or 28 or 29 or 30 or 31 or 32 or 33 or 34 or 35 or 36 or 37 or 38 or 39 | 480067 |
|  | 43 | 40 and 41 and 42 | 9807 |
|  | 44 | limit 43 to yr="2011 -Current" | **5,844** |
