# Supplementary material for: Health equity for persons with disabilities: a global scoping review on barriers and interventions in healthcare services
Source: Int J Equity Health. 2023 Nov 13;22:236. doi: 10.1186/s12939-023-02035-w (PMC10644565; doi:10.1186/s12939-023-02035-w)
Supplement: Supplementary file 3 — Additional file 3. Characteristics of the included studies. [file 12939_2023_2035_MOESM3_ESM.docx]

## Additional file 3: Characteristics of the included articles

***Table 1:*** *Characteristics of the included articles on the barriers of access to healthcare services for persons with disabilities (RQ1)*

| **#** | **Authors** | **Year** | **Country** | **WHO Region^1^** | **Level of income^2^** | **Methodology** | **Disability^3^** | **Perspectives^4^** | **Sample size** | **Intersectionality^5^** | **Healthcare service** | **Barriers^6^** | | | | | | | | |
| --- | --- | --- | --- | --- | --- | --- | --- | --- | --- | --- | --- | --- | --- | --- | --- | --- | --- | --- | --- | --- |
|  |  |  |  |  |  |  |  |  |  |  |  | **Health systems** | | | | | | **SF** | **SDoH** | **RF** |
|  |  |  |  |  |  |  |  |  |  |  |  | **W** | **I** | **F** | **L** | **D** | **E** |  |  |  |
| (1) | Adigun et al. | 2021 | Global | – | – | Systematic review (n=6) | Sensory | Persons with disabilities | n=2,105 | *Women* | Maternal care | + | – | – | – | + | – | – | + | + |
| (2) | Aenishanslin et al. | 2020 | Sierra Leone | AFRO | Low | Qualitative | Physical | Persons with disabilities | n=38 | – | Rehabilitation | + | – | + | + | + | + | + | + | – |
| (3) | Agaronnik et al. | 2021 | USA | PAHO | High | Qualitative | Physical | Persons with disabilities | n=20 | – | Cancer care | + | – | – | – | – | + | – | – | – |
| (4) | Alduhaim et al. | 2020 | Kuwait | EMRO | High | Qualitative | Sensory | Caregivers of children with disabilities | n=21 | *Children* | Rehabilitation | + | – | – | – | + | + | – | – | – |
| (5) | Allen et al. | 2020 | Sierra Leone | AFRO | Low | Mixed methods | Physical | Persons with disabilities; healthcare workers | n=21 | – | Rehabilitation | – | – | + | + | + | + | + | + | – |
| (6) | Arulogun et al. | 2013 | Nigeria | AFRO | Lower middle | Quantitative | Sensory | Persons with disabilities | n=167 | *Women + Children* | Sexual and reproductive healthcare | + | – | + | – | + | – | – | + | – |
| (7) | Bay et al. | 2016 | Norway | EURO | High | Qualitative | Psychosocial | Persons with disabilities | n=8 | – | Mental health care | + | – | – | – | + | – | + | + | + |
| (8) | Bernal et al. | 2019 | USA | PAHO | High | Quantitative | Mixed | Persons with disabilities | n=9,406 | – | Healthcare | + | + | + | – | + | – | – | – | – |
| (9) | Borade et al. | 2021 | India | SEARO | Lower middle | Qualitative | Physical | Persons with disabilities | n=25 | – | Rehabilitation | + | + | + | + | + | + | + | – | – |
| (10) | Bradbury-Jones et al. | 2015 | UK | EURO | High | Qualitative | Mixed | Healthcare workers | n=45 | *Women* | Maternal care | + | – | – | + | + | – | + | + | + |
| (11) | Bradbury-Jones et al. | 2015 | UK | EURO | High | Qualitative | Mixed | Persons with disabilities | n=5 | *Women* | Maternal care | + | – | – | – | + | + | + | + | – |
| (12) | Breckenridge et al. | 2014 | Global | – | – | Systematic review (n=11) | Mixed | Persons with disabilities; healthcare workers | – | *Women* | Maternal care | + | – | + | + | + | + | + | + | – |
| (13) | Bright et al. | 2018 | Global | – | Low, middle | Systematic review (n=77) | Mixed | – | – | – | Rehabilitation | + | – | + | – | + | + | + | + | + |
| (14) | Bryant et al. | 2021 | Australia | WPRO | High | Qualitative | Psychosocial | Healthcare workers | n=16 | *Elderly + Ethnicity* | Dementia care | + | – | + | – | + | + | + | + | + |
| (15) | Cabral et al. | 2013 | USA | PAHO | High | Qualitative | Sensory | Persons with disabilities | n=12 | – | Mental health care | + | – | – | – | + | – | + | + | – |
| (16) | Cassell et al. | 2012 | USA | PAHO | High | Mixed methods | Physical | Caregivers | n=248 | *Children* | Healthcare | + | + | + | – | + | + | + | + | – |
| (17) | Coleman-Fountain et al. | 2020 | UK | EURO | High | Qualitative | Intellectual | Persons with disabilities (autism) | n=19 | – | Mental health care | + | – | – | – | + | – | – | – | + |
| (18) | DeBeaudrap et al. | 2019 | Cameroon | AFRO | Lower middle | Quantitative | Mixed | Persons with disabilities and without disabilities | n=620 | – | Sexual and reproductive healthcare | + | – | + | – | + |  | – | – | – |
| (19) | Deng et al. | 2021 | China | WPRO | Upper middle | Qualitative | Psychosocial | Healthcare workers | n=36 | *Rural* | Mental health care | + | – | + | + | + | – | + | + | + |
| (20) | Dockery et al. | 2015 | UK | EURO | High | Mixed methods | Psychosocial | Persons with disabilities; caregivers | n=282 | – | Mental health care | – | – | + | – | + | – | + | + | + |
| (21) | Ee et al. | 2021 | Singapore | WPRO | High | Qualitative | Intellectual | Healthcare workers | n=8 | – | Mental health care | + | – | – | – | + | – | + | + | – |
| (22) | Elpers et al. | 2016 | USA | PAHO | High | Qualitative | Sensory | Caregivers of children with disabilities | n=40 | *Children + Rural* | Rehabilitation | + | + | + | – | + | – | – | + | – |
| (23) | Gallego et al. | 2017 | Australia | WPRO | High | Mixed methods | Mixed | Caregivers | n=166 | *Rural* | Rehabilitation | + | – | + | – | + | + | – | + | – |
| (24) | Ganle et al. | 2016 | Ghana | AFRO | Lower middle | Qualitative | Mixed | Persons with disabilities | n=72 | *Women* | Maternal care | + | – | + | – | + | + | + | + | – |
| (25) | Gibbons et al. | 2016 | USA | PAHO | High | Qualitative | Intellectual | Persons with disabilities | n=23 | – | Healthcare | + | + | + | – | + | + | – | + | – |
| (26) | Gormez et al. | 2020 | Turkey | EURO | Upper middle | Mixed methods | Intellectual | Persons with disabilities and their caregivers | n=771 | – | Mental health care | – | – | – | – | + | – | – | + | – |
| (27) | Green et al. | 2020 | USA | PAHO | High | Mixed methods | Psychosocial | Persons with disabilities (n=93); healthcare workers | n=107 | *Elderly* | Healthcare | + | + | – | – | + | – | – | + | + |
| (28) | Guerin et al. | 2017 | New Zealand | WPRO | High | Mixed methods | Mixed | Persons with disabilities | n=20 | *Women* | Maternal care | + | + | + | – | + | + | – | + | – |
| (29) | Guzder et al. | 2013 | Canada | PAHO | High | Qualitative | Psychosocial | Caregivers of children with disabilities | n=20 | *Children + Immigrants* | Mental health care | – | + | – | – | + | – | + | + | – |
| (30) | Harris et al. | 2016 | Australia | WPRO | High | Quantitative | Psychosocial | Persons with disabilities | n=1,764 | *Women* | Mental health care | – | – | + | – | + | – | + | + | – |
| (31) | Hasan et al. | 2017 | Jordan | EMRO | Upper middle | Qualitative | Psychosocial | Caregivers | n=27 | – | Mental health care | – | – | + | – | + | – | + | + | – |
| (32) | Henderson et al. | 2011 | Australia | WPRO | High | Qualitative | Psychosocial | Stakeholders | n=10 | – | Healthcare | + | + | + | + | + | – | + | + | – |
| (33) | Hill et al. | 2020 | Global | – | – | Systematic review (n=34) | Sensory | – | – | – | Cancer care | + | – | – | – | + | – | + | + | – |
| (34) | Horner-Johnson et al. | 2021 | USA | PAHO | High | Qualitative | Mixed | Persons with disabilities | n=17 | *Women* | Sexual and reproductive healthcare | + | – | + | – | + | + | – | – | – |
| (35) | Igwesi-Chidobe et al. | 2012 | Nigeria | AFRO | Lower middle | Mixed methods | Physical | Persons with and without disabilities; stakeholders | n=424 | *Rural* | Rehabilitation | + | + | + | + | + | + | – | + | – |
| (36) | Jindal et al. | 2018 | India / Canada | SEARO / PAHO | Lower middle / High | Qualitative | Intellectual | Caregivers of children with disabilities | n=18 | *Children* | Rehabilitation | + | + | + | – | + | – | – | + | – |
| (37) | Kaufman et al. | 2012 | USA | PAHO | High | Qualitative | Psychosocial | Persons with disabilities; healthcare workers | n=30 | – | Healthcare | + | + | + | – | + | – | + | + | + |
| (38) | Kenya Union of the Blind & World Blind Union | 2020 | Kenya | AFRO | Lower middle | Mixed methods | Mixed | Persons with disabilities; stakeholders | n=136 | – | Healthcare | + | – | + | – | + | + | – | + | – |
| (39) | Kumurenzi et al. | 2015 | South Africa | AFRO | Upper middle | Qualitative | Physical | Persons with disabilities; healthcare workers | n=14 | – | Rehabilitation | + | – | – | – | + | + | – | + | – |
| (40) | Lee et al. | 2017 | Australia | WPRO | High | Qualitative | Psychosocial | Persons with disabilities; healthcare workers | n=59 | – | Rehabilitation | + | + | + | + | + | – | – | + | + |
| (41) | Lewis et al. | 2016 | UK | EURO | High | Qualitative | Intellectual | Persons with disabilities | n=6 | – | Mental health care | – | – | – | – | + | – | – | – | – |
| (42) | Lindsay et al. | 2012 | Canada | PAHO | High | Qualitative | Mixed | Healthcare workers; stakeholders | n=13 | *Children + Immigrants* | Rehabilitation | + | – | – | – | + | – | + | + | – |
| (43) | Makhoba et al. | 2016 | South Africa | AFRO | Upper middle | Mixed methods | Sensory | Healthcare workers | n=45 | – | Rehabilitation | + | – | + | – | – | – | – | – | – |
| (44) | Man et al. | 2019 | Australia | WPRO | High | Quantitative | Intellectual & Psychosocial | Caregivers | n=41 | – | Mental health care | + | – | + | – | – | – | – | + | – |
| (45) | Marthoenis et al. | 2016 | Indonesia | SEARO | Lower middle | Qualitative | Psychosocial | Caregivers | n=16 | – | Mental health care | – | – | + | – | + | – | + | + | – |
| (46) | McBain et al. | 2016 | UK | EURO | High | Qualitative | Psychosocial | Healthcare workers | n=16 | – | Healthcare | + | + | – | – | + | – | – | – | + |
| (47) | Mimmo et al. | 2019 | Australia, Norway, Switzerland, USA, Sweden, Canada, UK | WPRO, EURO, PAHO | High | Meta-narrative of qualitative studies (n=11) | Intellectual | Caregivers; healthcare workers | n=603 | *Children* | Healthcare | + | – | – | – | – | – | – | – | – |
| (48) | Mitchell et al. | 2021 | Australia, New Zealand | WPRO | High | Mixed methods | Psychosocial | Healthcare workers | n=24 | *Elderly* | Rehabilitation | + | – | + | – | + | + | + | – | – |
| (49) | Mitra et al. | 2017 | USA | PAHO | High | Mixed methods | Physical | Persons with disabilities | n=126 | *Women* | Maternal care | + | – | – | – | + | + | – | – | – |
| (50) | Mkabile et al. | 2021 | South Africa | AFRO | Upper middle | Qualitative | Intellectual | Caregivers | n=26 | *Children* | Healthcare | – | – | + | – | – | – | + | + | – |
| (51) | Morris et al. | 2014 | USA | PAHO | High | Qualitative | Mixed | Persons with disabilities | n=12 | – | Healthcare | + | – | – | – | – | – | – | – | – |
| (52) | Murphy et al. | 2021 | USA | PAHO | High | Mixed methods | Psychosocial | Healthcare workers | n=32 | – | Cancer care | + | – | + | – | + | – | – | + | + |
| (53) | National Council on Disability | 2019 | USA | PAHO | High | Mixed methods | Mixed | Persons with disabilities | – | – | Healthcare | + | – | – | + | – | – | – | – | – |
| (54) | National Council on Disability | 2021 | USA | PAHO | High | Mixed methods | Mixed | Persons with disabilities | – | – | Healthcare | + | + | – | + | + | + | + | + | – |
| (55) | Nguyen et al. | 2019 | Vietnam | WPRO | Lower middle | Qualitative | Physical | Persons with disabilities | n=20 | – | Sexual and reproductive healthcare | + | – | + | – | + | + | + | + | – |
| (56) | Novak et al. | 2019 | USA | PAHO | High | Quantitative | Psychosocial | Persons with disabilities | n=3,398 | – | Mental health care | – | – | + | – | + | – | + | – | – |
| (57) | Nuri et al. | 2021 | Bangladesh | SEARO | Lower middle | Qualitative | Mixed | Healthcare workers for children with disabilities | n=21 | *Children* | Rehabilitation | + | + | + | + | + | + | + | + | – |
| (58) | O’Halloran et al. | 2012 | Australia | WPRO | High | Qualitative | Mixed | Persons with disabilities with healthcare workers | n=65 | – | Healthcare (stroke units) | + | – | – | – | + | + | – | – | – |
| (59) | Oyelade et al. | 2021 | Nigeria | AFRO | Lower middle | Qualitative | Psychosocial | Persons with disabilities | n=29 | – | Mental health care | – | – | + | – | + | – | + | + | – |
| (60) | Pearson et al. | 2020 | New Zealand | WPRO | High | Mixed methods | Mixed | Persons with disabilities | n=84 | *Women* | Cancer care | + | + | + | + | + | + | + | + | – |
| (61) | Peters et al. | 2015 | Australia | WPRO | High | Qualitative | Physical | Persons with disabilities | n=12 | *Women* | Cancer care | + | – | – | – | + | – | – | + | – |
| (62) | Pitman et al. | 2011 | UK | EURO | High | Quantitative | Psychosocial | Persons with disabilities; healthcare workers | n=536 | – | Healthcare | + | + | + | – | + | + | – | + | + |
| (63) | Poon et al. | 2013 | Australia | WPRO | High | Qualitative | Psychosocial | Caregivers; healthcare workers | n=12 | *Migrants* | Mental health care | + | – | – | – | + | + | + | + | – |
| (64) | Porat et al. | 2012 | Israel | EURO | High | Quantitative | Physical & Sensory | – | n=1,203 | *Children* | Sexual and reproductive healthcare | – | – | – | – | – | – | + | + | – |
| (65) | Redfern et al. | 2016 | Australia | WPRO | High | Mixed methods | Mixed | Healthcare workers | n=5 | – | Healthcare | – | + | + | + | + | – | – | – | + |
| (66) | Relyea et al. | 2019 | Canada | PAHO | High | Scoping review (n=32) | Psychosocial | – | – | – | Healthcare (palliative care) | + | + | – | – | + | – | – | + | + |
| (67) | Roux-Levy et al. | 2021 | France | EURO | High | Mixed Methods | Intellectual | Persons with disabilities; healthcare workers | n=108 | – | Healthcare | – | – | + | – | + | – | – | – | – |
| (68) | Sakellariou et al. | 2017 | UK | EURO | High | Quantitative | Mixed | Persons with disabilities | n=5,230 | *Women* | Healthcare | – | – | + | – | + | – | – | + | – |
| (69) | Saleeby et al. | 2016 | USA | PAHO | High | Qualitative | Mixed | Persons with disabilities | n=10 | *Women* | Cancer care | + | – | + | + | + | + | – | + | – |
| (70) | Samtani et al. | 2021 | UK / Canada / USA / France / Australia | EURO / PAHO / WPRO | High | Scoping review (n=12) | Mixed | – | – | – | Cancer care | + | + | – | + | + | – | – | – | + |
| (71) | Schildberger et al. | 2017 | Austria | EURO | High | Qualitative | Mixed | Persons with disabilities | n=10 | *Women* | Maternal care | + | – | – | – | + | – | + | + | – |
| (72) | SightSavers | 2014 | Sierra Leone | AFRO | Low | Mixed Methods | Sensory | Persons with and without disabilities; caregivers; stakeholders | n=1,099 | – | Eye care | – | – | + | – | + | – | + | + | – |
| (73) | Silva et al. | 2020 | Brazil | PAHO | Upper middle | Quantitative | Physical | Caregivers of children with disabilities; healthcare workers | n=168 | *Children* | Rehabilitation | + | – | + | – | + | – | + | + | – |
| (74) | Smeltzer et al. | 2018 | USA | PAHO | High | Qualitative | Physical | Healthcare workers | n=14 | *Women* | Maternal care | + | + | + | – | + | – | + | – | – |
| (75) | Tabril et al. | 2020 | Morocco | EMRO | Lower middle | Quantitative | Psychosocial | Healthcare workers | n=402 | – | Healthcare | + | – | + | – | + | + | – | + | + |
| (76) | Tarasoff et al. | 2017 | Canada | PAHO | High | Qualitative | Physical | Persons with disabilities | n=13 | *Women* | Maternal care | + | + | – | – | + | + | + | + | + |
| (77) | Tuffrey-Wijne et al. | 2014 | UK | EURO | High | Mixed Methods | Intellectual | Persons with disabilities, caregivers, stakeholders | n=1,258 | – | Healthcare | + | + | + | + | – | – | – | – | – |
| (78) | UNICEF | 2017 | India | SEARO | Lower middle | Qualitative | Mixed | Caregivers, stakeholders | n=1,128 | *Children* | Healthcare | + | + | + | + | + | – | + | + | – |
| (79) | Voillemont et al. | 2021 | France | EURO | High | Qualitative | Intellectual | Healthcare workers | n=11 | – | Healthcare | + | + | – | – | + | + | – | + | + |
| (80) | Webb et al. | 2011 | UK | EURO | High | Mixed Methods | Mixed | Caregivers of children with disabilities | n=405 survey; n=9 interviews | *Children* | Healthcare | + | + | – | – | + | – | – | – | – |
| (81) | Wheeler et al. | 2019 | USA | PAHO | High | Quantitative | Intellectual | Caregivers of children with disabilities | n=731 | *Children* | Healthcare | + | – | + | – | + | – | – | – | – |
| (82) | Wong et al. | 2019 | USA | PAHO | High | Quantitative | Physical | Persons with disabilities | n=1,159 | – | Healthcare | – | – | + | – | + | + | – | + | – |
| (83) | Zuurmond | 2019 | Cameroon / India | AFRO / SEARO | Lower middle | Qualitative | Mixed | Persons with disabilities; stakeholders | n=91 | – | Healthcare | + | + | + | + | + | + | + | + | + |
| ***^1^ WHO Regions:*** *The six WHO world regions are (1) African Region; (2) Region of the Americas; (3) South-East Asia Region; (4) European Region; (5) Eastern Mediterranean Region; (6) Western Pacific Region. More details available on the WHO website (84).*  ***^2^ Level of income:*** *The level of income indicated for each country follows the classification produced by the World Bank: (1) Low-income; (2) Lower middle income; (3) Upper middle income; (4) High-income. More details on the World Bank website (85).*  ***^3^ Disability:*** *The classification of disability types indicated in this column is aligned with the United Nations Convention on the Rights of Persons with Disabilities (UNCRPD): (1) Physical; (2) Psychosocial; (3) Intellectual; and (4) Sensory. More details on the United Nations’ Department of Economic and Social Affairs’ website (86).*  ***^4^ Perspectives:*** *This column indicates the profile of individuals and groups who reported the barriers of access to healthcare services for persons with disabilities.*  ***^5^ Intersectionality:*** *This column indicates whether the articles focused on intersectional identities which can lead to unique experiences of health and an increased risk of health disparities for certain groups of persons with disabilities. To extract information on intersectionality, we consulted the UN list of vulnerable groups (e.g., women, LGBTQI+, race, or migrants) and complemented it with the multiple identities of persons with disabilities elaborated in the UNCRPD (e.g., children with disabilities) (87-89). Most notably, this included children, women, elderly or immigrants with disabilities, or persons with disabilities with minoritized ethnic backgrounds or living in rural areas.*  ***^6^ Barriers:*** *The barriers are structured according to the building blocks of health systems (Health and care workforce (W); Health information systems (I); Health systems financing (F); Leadership and governance (L); Service delivery (D); Essential medicines & equipment (E)) and other factors (Structural factors (SF); Social determinants of health (SDoH); Risk factors (RF)).*  ***Legend:*** *The “+” sign indicates that we found and extracted evidence on this category of barriers from the article. The “–“ sign indicates that we did not find and extract evidence on this category of barriers from the article.* | | | | | | | | | | | | | | | | | | | | |

***Table 2:*** *Characteristics of the included articles on the interventions of access to healthcare services for persons with disabilities (RQ2)*

| **#** | **Authors** | **Year** | **Country** | **WHO Region^1^** | **Level of income^2^** | **Methodology** | **Disability^3^** | **Intersectionality^4^** | **Intervention type** | **Intervention’s primary focus^5^** | | | | | | | | | **Scaled up** |
| --- | --- | --- | --- | --- | --- | --- | --- | --- | --- | --- | --- | --- | --- | --- | --- | --- | --- | --- | --- |
|  |  |  |  |  |  |  |  |  |  | **Health systems** | | | | | | **SF** | **SDoH** | **RF** |  |
|  |  |  |  |  |  |  |  |  |  | **W** | **I** | **F** | **L** | **D** | **E** |  |  |  |  |
| (90) | Alduhaim et al. | 2021 | Kuwait | EMRO | High | Quantitative | Sensory | *Children* | Education | *+* | – | – | – | – | – | – | – | – | No |
| (91) | Amin et al. | 2020 | USA | PAHO | High | Mixed methods | Psychosocial | – | Education | *+* | – | – | – | – | – | – | – | – | No |
| (92) | Archibald et al. | 2018 | Canada | PAHO | High | Mixed methods | Psychosocial | – | Telehealth | – | + | – | – | – | – | – | – | – | Yes (regional) |
| (93) | Armstrong et al. | 2016 | – | – | – | Systematic review (n=12) | Mixed | *Children* | Education | – | – | – | – | – | – | + | – | – | – |
| (94) | Aviram et al. | 2012 | Israel | EURO | High | Qualitative | Psychosocial | – | Policy | – | – | – | + | – | – | – | – | – | Yes (national) |
| (95) | Barr et al. | 2021 | Australia | WPRO | High | Mixed methods | Sensory | *Children* | Health insurance | – | – | + | – | – | – | – | – | – | Yes (national) |
| (96) | Battistella et al. | 2015 | Brazil | PAHO | Upper middle | Mixed methods | Physical | – | Community outreach | – | – | – | – | + | – | – | – | – | Yes (national) |
| (97) | Breslau et al. | 2021 | USA | PAHO | High | Quantitative | Psychosocial | – | Task-sharing | – | – | – | – | + | – | – | – | – | Yes (national) |
| (98) | Broussard et al. | 2014 | USA | PAHO | High | Qualitative | Psychosocial | *Ethnicity* | Education | – | – | – | – | – | – | – | + | – | No |
| (99) | Brown et al. | 2016 | USA | PAHO | High | Mixed methods | Mixed | *Children* | Quality care | – | – | – | – | + | – | – | – | – | No |
| (100) | Buchan et al. | 2020 | Global | Mixed | High | Systematic review (n=11) | Psychosocial | – | Quality care (participation) | – | – | – | – | + | – | – | – | – | No |
| (101) | Buszewicz et al. | 2014 | UK | EURO | High | Quantitative | Intellectual | – | Education & Quality care | + | – | – | – | + | – | – | – | – | Yes (national) |
| (102) | Byrne et al. | 2013 | Australia | WPRO | High | Qualitative | Psychosocial | – | Education | + | – | – | – | – | – | – | – | – | No |
| (103) | Callanan et al. | 2021 | Australia | WPRO | High | Quantitative | Mixed | *Children* | Education | + | – | – | – | – | – | – | + | – | No |
| (104) | Chien et al. | 2012 | China | WPRO | Upper middle | Quantitative | Psychosocial | – | Task-sharing | – | – | – | – | + | – | – | – | – | No |
| (105) | Collins et al. | 2019 | USA | PAHO | High | Quantitative | Psychosocial | – | Education | – | – | – | – | – | – | + | – | – | Yes (national) |
| (106) | Cook et al. | 2013 | USA | PAHO | High | Quantitative | Psychosocial | – | Quality care & Education | – | – | – | – | + | – | – | + | – | No |
| (107) | Corrigan et al. | 2018 | USA | PAHO | High | Quantitative | Psychosocial | *Ethnicity* | Quality care & Education | – | – | – | – | + | – | – | + | – | No |
| (108) | Crowley et al. | 2011 | – | – | – | Systematic review (n=10) | Mixed | *Children* | Care coordination | – | – | – | – | + | – | – | – | – | – |
| (109) | D’Aprano et al. | 2020 | Australia | WPRO | High | Mixed methods | Mixed | *Children* | Care coordination | – | – | – | – | + | – | – | – | – | No |
| (110) | Dagnan et al. | 2018 | UK | EURO | High | Quantitative | Intellectual | – | Education | + | – | – | – | – | – | – | – | – | No |
| (111) | Devine et al. | 2017 | Philippines | WPRO | Lower middle | Qualitative | Mixed | *Women* | Quality care (participation) & Education | – | – | – | – | + | – | + | + | – | Yes (urban) |
| (112) | du Toit et al. | 2017 | – | – | Low and middle | Systematic review (n=31) | Sensory | *Children* | Task-sharing | – | – | – | – | + | – | – | – | – | – |
| (113) | Fiander et al. | 2012 | Tanzania | AFRO | Lower middle | Quantitative | Physical | – | Transport | – | – | – | – | – | – | – | + | – | Yes (national) |
| (114) | Evans-Lacko et al. | 2013 | UK | EURO | High | Quantitative | Psychosocial | – | Education | – | – | – | – | – | – | + | – | – | Yes (national) |
| (115) | Feldman et al. | 2012 | Canada | PAHO | High | Quantitative | Intellectual | – | Education | – | – | – | – | – | – | – | + | – | Yes (regional) |
| (116) | Giesbrecht et al. | 2014 | Canada | PAHO | High | Qualitative | Physical | *Elderly* | Telehealth | – | – | – | – | + | – | – | – | – | No |
| (117) | Gillies et al. | 2015 | – | – | – | Systematic review (n=12) | Psychosocial | – | Education & Task-sharing | + | – | – | – | – | – | – | – | – | – |
| (118) | Golyk et al. | 2021 | Ukraine | EURO | Lower middle | Qualitative | Mixed | – | Policy | – | – | – | + | – | – | – | – | – | Yes (national) |
| (119) | Grady et al. | 2011 | USA | PAHO | High | Quantitative | Psychosocial | *Rural* | Telehealth | – | – | – | – | + | – | – | – | – | No |
| (120) | Gureje et al. | 2020 | Nigeria, Ghana | AFRO | Lower middle | Quantitative | Psychosocial | – | Multisectoral coordination | – | – | – | – | + | – | – | – | – | No |
| (121) | Hamblen et al. | 2019 | USA | PAHO | High | Quantitative | Psychosocial | – | Telehealth | – | – | – | – | – | – | + | – | – | Yes (–) |
| (122) | Hamdani et al. | 2014 | Canada | PAHO | High | Qualitative | Mixed | *Children* | Care coordination | – | – | – | – | + | – | – | – | – | No |
| (123) | Hammarberg et al. | 2014 | Australia | WPRO | High | Qualitative | Mixed | *Children* | Caregiver support | – | – | – | – | + | – | – | + | – | Yes (national) |
| (124) | Happell et al. | 2014 | – | – | – | Systematic review (n=30) | Psychosocial | – | Education | + | – | – | – | – | – | – | – | – | – |
| (125) | Harney et al. | 2021 | Australia | WPRO | High | Quantitative | Psychosocial | – | Care coordination | – | – | – | – | + | – | – | – | – | No |
| (126) | Henderson et al. | 2014 | – | – | – | Review (–) | Psychosocial | – | Education | + | – | – | – | – | – | – | – | – | – |
| (127) | Hensley et al. | 2011 | USA | PAHO | High | Qualitative | Psychosocial | – | Funding | – | – | + | – | – | – | – | – | – | Yes |
| (128) | Highland et al. | 2020 | USA | PAHO | High | Quantitative | Psychosocial | – | Care coordination | – | – | – | – | + | – | – | – | – | No |
| (129) | Irwin et al. | 2019 | USA | PAHO | High | Quantitative | Psychosocial | – | Care coordination | – | – | – | – | + | – | – | – | – | No |
| (130) | Isaacs et al. | 2017 | Australia | WPRO | High | Qualitative | Psychosocial | – | Multisectoral coordination | – | – | – | – | + | – | – | – | – | Yes (district) |
| (131) | Johnson et al. | 2021 | USA | PAHO | High | Quantitative | Psychosocial | – | Care coordination | – | – | – | – | + | – | – | – | – | No |
| (132) | Jokel et al. | 2017 | Canada | PAHO | High | Quantitative | Mixed | – | Education | – | – | – | – | – | – | – | + | – | No |
| (133) | Jones et al. | 2016 | Australia | WPRO | High | Qualitative | Psychosocial | – | Multisectoral coordination; Education | – | – | – | – | + | – | – | – | – | Yes (regional) |
| (134) | Kennedy et al. | 2017 | USA | PAHO | High | Quantitative | Mixed | – | Policy | – | – | + | + | – | – | – | – | – | Yes (national) |
| (135) | Kilbourne et al. | 2015 | USA | PAHO | High | Quantitative | Psychosocial | – | Education | + | – | – | – | – | – | – | – | – | Yes (national) |
| (136) | King et al. | 2018 | Cambodia | WPRO | Lower middle | Qualitative | Mixed | – | Transport | – | – | – | – | – | – | – | + | – | No |
| (137) | Kranz et al. | 2020 | USA | PAHO | High | Quantitative | Intellectual | *Children* | Policy | – | – | – | + | – | – | – | – | – | Yes (national) |
| (138) | Kreutzberg et al. | 2020 | UK | EURO | High | Quantitative | Psychosocial | – | Quality care | – | – | – | – | + | – | – | – | – | Yes (national) |
| (139) | Lam et al. | 2020 | Canada | PAHO | High | Quantitative | Psychosocial | – | Care coordination; Task-sharing | – | – | – | – | + | – | – | – | – | No |
| (140) | Li et al. | 2019 | China | WPRO | Upper middle | Quantitative | Psychosocial | – | Education | + | – | – | – | – | – | – | – | – | No |
| (141) | Lovero et al. | 2019 | South Africa | AFRO | Upper middle | Mixed methods | Psychosocial | *Women* | Policy; Care coordination | – | – | – | + | + | – | – | – | – | Yes (district) |
| (142) | MacDonald et al. | 2018 | UK | EURO | High | Qualitative | Intellectual | – | Task-sharing | – | – | – | – | + | – | – | – | – | No |
| (143) | Mackinnon et al. | 2016 | UK | EURO | High | Qualitative | Psychosocial | – | Quality care | – | – | – | – | + | – | – | – | – | Yes (national) |
| (144) | Malik et al. | 2012 | – | – | – | Systematic review (n=26) | Psychosocial | – | Education | + | – | – | – | – | – | – | – | – | – |
| (145) | Martin-Prudent et al. | 2016 | USA | PAHO | High | Quantitative | Sensory | *Children* | Education; Care coordination | + | – | – | – | + | – | – | – | – | No |
| (146) | Mathias et al. | 2018 | India | SEARO | Lower middle | Qualitative | Psychosocial | – | Multisectoral coordination; Education | – | – | – | – | + | – | + | – | – | Yes (district) |
| (147) | McClellan et al. | 2020 | USA | PAHO | High | Quantitative | Psychosocial | – | Insurance; Policy; Care coordination | – | – | + | + | + | – | – | – | – | Yes (national) |
| (148) | Mehta et al. | 2015 | – | – | – | Systematic review (n=80) | Psychosocial | – | Education | – | – | – | – | – | – | + | – | – | – |
| (149) | Mejia-Lancheros et al. | 2021 | Canada | PAHO | High | Quantitative | Psychosocial | – | Multisectoral coordination | – | – | – | – | + | – | – | + | – | Yes (national) |
| (150) | Mittal et al. | 2012 | – | – | – | Systematic review (n=14) | Psychosocial | – | Education | – | – | – | – | – | – | – | + | – | – |
| (151) | Morabito et al. | 2018 | USA | PAHO | High | Mixed methods | Psychosocial | – | Multisectoral coordination | – | – | – | – | + | – | + | + | – | No |
| (152) | Morriss et al. | 2013 | – | – | – | Systematic review (n=32) | Psychosocial | – | Education | + | – | – | – | – | – | – | + | – | – |
| (153) | Moxham et al. | 2017 | Australia | WPRO | High | Qualitative | Psychosocial | – | Education | + | – | – | – | – | – | – | – | – | No |
| (154) | Mudrick et al. | 2020 | USA | PAHO | High | Qualitative | Mixed | – | Healthcare information; Quality care | – | + | – | – | + | – | – | – | – | No |
| (155) | Mueller-Stierlin | 2017 | Germany | EURO | High | Quantitative | Psychosocial | – | Multisectoral coordination; Quality care; Insurance | – | – | + | – | + | – | – | – | – | Yes (regional) |
| (156) | Murphy et al. | 2018 | USA | PAHO | High | Systematic review (n=11) | Psychosocial | – | Care coordination | – | – | – | – | + | – | – | – | – | – |
| (157) | Musyimi et al. | 2016 | Kenya | AFRO | Lower middle | Qualitative | Psychosocial | – | Multisectoral coordination | – | – | – | – | + | – | + | – | – | No |
| (158) | Neherta et al. | 2019 | Indonesia | WPRO | Lower middle | Quantitative | Intellectual | *Children* | Education | – | – | – | – | – | – | – | + | – | No |
| (159) | O’Donovan et al. | 2020 | Uganda | AFRO | Low | Qualitative | Sensory | – | Education; Task-sharing; Multisectoral coordination | + | – | – | – | – | – | + | – | – | No |
| (160) | Oliveira et al. | 2016 | Brazil | PAHO | Upper middle | Quantitative | Sensory | – | Accessible health information | – | – | – | – | – | – | – | + | – | No |
| (161) | Oliveira et al. | 2018 | Brazil | PAHO | Upper middle | Quantitative | Sensory | *Women* | Accessible health information | – | – | – | – | – | – | – | + | – | No |
| (162) | Owen et al. | 2020 | USA | PAHO | High | Quantitative | Mixed | – | Care coordination | – | – | – | – | + | – | – | – | – | Yes (national) |
| (163) | Parish et al. | 2012 | USA | PAHO | High | Quantitative | Mixed | *Children* | Policy; Insurance; Quality care | – | – | + | + | + | – | – | + | – | Yes (national) |
| (164) | Pathare et al. | 2021 | India | SEARO | Lower middle | Quantitative | Psychosocial | – | Quality care | – | – | – | – | + | – | + | – | – | Yes (regional) |
| (165) | Pennybaker et al. | 2016 | – | – | – | Systematic review (n=49) | Psychosocial | – | Multisectoral coordination | – | – | – | – | + | – | + | – | – | – |
| (166) | Poltorak et al. | 2016 | Tonga | WPRO | Upper middle | Qualitative | Psychosocial | – | Multisectoral coordination | – | – | – | – | + | – | + | + | – | Yes (national) |
| (167) | Porter et al. | 2012 | UK | EURO | High | Qualitative | Intellectual | *Women* | Accessible health information | – | – | – | – | – | – | – | + | – | No |
| (168) | Rai et al. | 2018 | Nepal | SEARO | Lower middle | Qualitative | Psychosocial | – | Education | + | – | – | – | – | – | – | – | – | Yes (district) |
| (169) | Rico-Blázqueza et al. | 2021 | Spain | EURO | High | Quantitative | Mixed | – | Caregiver support | – | – | – | – | + | – | – | + | – | No |
| (170) | Ride et al. | 2018 | UK | EURO | High | Quantitative | Psychosocial | – | Quality care | – | – | – | – | + | – | – | – | – | Yes (national) |
| (171) | Robles-Bykbaev et al. | 2019 | Ecuador | PAHO | Upper middle | Quantitative | Sensory | *Women* | Accessible health information | – | – | – | – | – | – | – | + | – | No |
| (172) | Röhricht et al. | 2021 | UK | EURO | High | Quantitative | Psychosocial | – | Telehealth | – | – | – | – | + | – | – | + | – | No |
| (173) | Romaire et al. | 2020 | USA | PAHO | High | Qualitative | Psychosocial | – | Care coordination; Insurance | – | – | + | – | + | – | – | – | – | Yes (national) |
| (174) | Rotenberg et al. | 2022 | – | – | – | Systematic review (n=78) | Mixed | – | Education | + | – | – | – | – | – | – | – | – | – |
| (175) | Schilling et al. | 2015 | Chile | PAHO | High | Quantitative | Psychosocial | – | Education | – | – | – | – | – | – | + | + | – | No |
| (176) | Shor et al. | 2015 | Israel | EURO | High | Qualitative | Psychosocial | – | Quality care | – | – | – | – | + | – | – | – | – | No |
| (177) | Smythe et al. | 2020 | – | – | Low and middle | Systematic review (n=20) | Mixed | *Children* | Education | – | – | – | – | – | – | + | + | – | – |
| (178) | Steinert et al. | 2016 | – | – | – | Systematic review (n=36) | Mixed | – | Legislation | – | – | – | + | – | – | – | – | – | – |
| (179) | Steele et al. | 2017 | USA | PAHO | High | Quantitative | Psychosocial | – | Care coordination | – | – | – | – | + | – | – | – | – |  |
| (180) | Thornicroft et al. | 2015 | – | – | – | Systematic review (–) | Psychosocial | – | Education | – | – | – | – | – | – | + | + | – | – |
| (181) | Tran et al. | 2012 | Australia | WPRO | High | Qualitative | Psychosocial | – | Accessible health information | – | – | – | – | – | – | – | + | – | Yes (national) |
| (182) | Trondsen et al. | 2018 | Norway | EURO | High | Qualitative | Psychosocial | – | Telehealth | – | – | – | – | + | – | – | – | – | No |
| (183) | Vaughan et al. | 2020 | Philippines | WPRO | Lower middle | Qualitative | Mixed | *Women* | Quality care (participation) & Education | – | – | – | – | + | – | + | + | – | Yes (urban) |
| (184) | Velligan et al. | 2016 | USA | PAHO | High | Mixed methods | Psychosocial | – | Care coordination | – | – | – | – | + | – | – | – | – | No |
| (185) | Willems et al. | 2021 | Rwanda | AFRO | Low | Quantitative | Psychosocial | – | Education | + | – | – | – | – | – | – | – | – | Yes (national) |
| (186) | Williams et al. | 2021 | Australia | WPRO | High | Qualitative | Psychosocial | – | Telehealth | – | – | – | – | + | – | – | – | – | – |
| (187) | Xiong et al. | 2015 | USA | PAHO | High | Quantitative | Psychosocial | – | Care coordination | – | – | – | – | + | – | – | – | – | No |
| (188) | Yanos et al. | 2015 | – | – | – | Systematic review (–) | Psychosocial | – | Education | – | – | – | – | – | – | + | + | – | – |
| ***^1^ WHO Regions:*** *The six WHO world regions are (1) African Region; (2) Region of the Americas; (3) South-East Asia Region; (4) European Region; (5) Eastern Mediterranean Region; (6) Western Pacific Region. More details available on the WHO website (84).*  ***^2^ Level of income:*** *The level of income indicated for each country follows the classification produced by the World Bank: (1) Low-income; (2) Lower middle income; (3) Upper middle income; (4) High-income. More details on the World Bank website (85).*  ***^3^ Disability:*** *The classification of disability types indicated in this column is aligned with the United Nations Convention on the Rights of Persons with Disabilities (UNCRPD): (1) Physical; (2) Psychosocial; (3) Intellectual; and (4) Sensory. More details on the United Nations’ Department of Economic and Social Affairs’ website (86).*  ***^4^ Intersectionality:*** *This column indicates whether the articles focused on intersectional identities which can lead to unique experiences of health and an increased risk of health disparities for certain groups of persons with disabilities. To extract information on intersectionality, we consulted the UN list of vulnerable groups (e.g., women, LGBTQI+, race, or migrants) and complemented it with the multiple identities of persons with disabilities elaborated in the UNCRPD (e.g., children with disabilities) (87-89). Most notably, this included children, women, elderly or immigrants with disabilities, or persons with disabilities with minoritized ethnic backgrounds or living in rural areas.*  ***^5^ Intervention’s primary focus:*** *The classification indicated in this column is structured according to the building blocks of health systems (Health and care workforce (W); Health information systems (I); Health systems financing (F); Leadership and governance (L); Service delivery (D); Essential medicines & equipment (E)) and other factors (Structural factors (SF); Social determinants of health (SDoH); Risk factors (RF)).*  ***Legend:*** *The “+” sign indicates that we found and extracted evidence that the intervention(s) mentioned in the article primarily targeted this aspect of the health system or wider contributing factors to health. The “–“ sign indicates that we did not find and extract evidence that the intervention(s) mentioned in the article primarily targeted this aspect of the health system or wider contributing factors to health.* | | | | | | | | | | | | | | | | | | | |

**References**

Uncategorized References

1. Adigun OT, Akinrinoye O, Obilor HN. Including the excluded in antenatal care: A systematic review of concerns for D/deaf pregnant women. Behavioral Sciences. 2021;11(5).

2. Aenishänslin J, Amara A, Magnusson L. Experiences accessing and using rehabilitation services for people with physical disabilities in Sierra Leone. Disability and Rehabilitation. 2020(9207179):1-10.

3. Agaronnik ND, El-Jawahri A, Kirschner K, Iezzoni LI. Exploring cancer treatment experiences for patients with preexisting mobility disability. American Journal of Physical Medicine & Rehabilitation. 2021;100(2):113-9.

4. Alduhaim A, Purcell A, Cumming S, Doble M. Parents' views about factors facilitating their involvement in the oral early intervention services provided for their children with hearing loss in Kuwait. International Journal of Pediatric Otorhinolaryngology. 2020;128.

5. Allen APT, Bolton WS, Jalloh MB, Halpin SJ, Jayne DG, Scott JDA. Barriers to accessing and providing rehabilitation after a lower limb amputation in Sierra Leone - A multidisciplinary patient and service provider perspective. Disability and Rehabilitation. 2020:1-8.

6. Arulogun OS, Titiloye MA, Afolabi NB, Oyewole OE, Nwaorgu OGB. Experiences of girls with hearing impairment in accessing reproductive health care services in Ibadan, Nigeria. African Journal of Reproductive Health. 2013;17(1):85-93.

7. Bay N, Bjørnestad J, Johannessen JO, Larsen TK, Joa I. Obstacles to care in first-episode psychosis patients with a long duration of untreated psychosis. Early Intervention in Psychiatry. 2016;10(1):71-6.

8. Bernal OA, McClintock HF, Kurichi JE, Kwong PL, Xie D, Streim JE, et al. Patient satisfaction and perceived quality of care among younger medicare beneficiaries according to activity limitation stages. Archives of Physical Medicine and Rehabilitation. 2019;100(2):289-99.

9. Borade N, Ingle A, Nagarkar A. Lived experiences of people with mobility-related disability using assistive devices. Disability and Rehabilitation: Assistive Technology. 2021;16(7):730-4.

10. Bradbury-Jones C, Breckenridge JP, Devaney J, Duncan F, Kroll T, Lazenbatt A, et al. Priorities and strategies for improving disabled women's access to maternity services when they are affected by domestic abuse: A multi-method study using concept maps. BMC Pregnancy and Childbirth. 2015;15.

11. Bradbury-Jones C, Breckenridge JP, Devaney J, Kroll T, Lazenbatt A, Taylor J. Disabled women's experiences of accessing and utilising maternity services when they are affected by domestic abuse: A critical incident technique study. BMC Pregnancy and Childbirth. 2015;15.

12. Breckenridge JP, Devaney J, Kroll T, Lazenbatt A, Taylor J, Bradbury-Jones C. Access and utilisation of maternity care for disabled women who experience domestic abuse: a systematic review. BMC Pregnancy and Childbirth. 2014;14.

13. Bright T, Wallace S, Kuper H. A systematic review of access to rehabilitation for people with disabilities in low- and middle-income countries. International Journal of Environmental Research and Public Health. 2018;15(10).

14. Bryant J, Noble N, Freund M, Rumbel J, Eades S, Sanson-Fisher R, et al. How can dementia diagnosis and care for Aboriginal and Torres Strait Islander people be improved? Perspectives of healthcare providers providing care in Aboriginal community controlled health services. BMC Health Services Research. 2021;21(1):699-.

15. Cabral L, Muhr K, Savageau J. Perspectives of people who are deaf and hard of hearing on mental health, recovery, and peer support. Community Mental Health Journal. 2013;49(6):649-57.

16. Cassell CH, Mendez DD, Strauss RP. Maternal perspectives: Qualitative responses about perceived barriers to care among children with orofacial clefts in North Carolina. The Cleft Palate-Craniofacial Journal. 2012;49(3):262-9.

17. Coleman-Fountain E, Buckley C, Beresford B. Improving mental health in autistic young adults: A qualitative study exploring help-seeking barriers in UK primary care. British Journal of General Practice. 2020;70(694):E356-E63.

18. DeBeaudrap P, Mouté C, Pasquier E, Mac-Seing M, Mukangwije PU, Beninguisse G. Disability and access to sexual and reproductive health services in Cameroon: A mediation analysis of the role of socioeconomic factors. International Journal of Environmental Research and Public Health. 2019;16(3):4-10.

19. Deng Y, Wang A-L, Frasso R, Ran M-S, Zhang T-M, Kong D, et al. Mental health-related stigma and attitudes toward patient care among providers of mental health services in a rural Chinese county. The International Journal of Social Psychiatry. 2021;68(3):610-8.

20. Dockery L, Jeffery D, Schauman O, Williams P, Ly SF, Bonnington O, et al. Stigma- and non-stigma-related treatment barriers to mental healthcare reported by service users and caregivers. Psychiatry Research. 2015;228(3):612-9.

21. Ee J, Kroese BS, Lim JM, Rose J. What do specialist mental health professionals think of the mental health services for people with intellectual disabilities in Singapore? Journal of Intellectual Disabilities. 2021;26(4):972-89.

22. Elpers J, Lester C, Shinn JB, Bush ML. Rural family perspectives and experiences with early infant hearing detection and intervention: A qualitative study. Journal of Community Health. 2016;41(2):226-33.

23. Gallego G, Dew A, Lincoln M, Bundy A, Chedid RJ, Bulkeley K, et al. Access to therapy services for people with disability in rural Australia: A carers' perspective. Health & Social Care in the Community. 2017;25(3):1000-10.

24. Ganle JK, Otupiri E, Obeng B, Edusie AK, Ankomah A, Adanu R. Challenges women with disability face in accessing and using maternal healthcare services in Ghana: A qualitative study. PLOS One. 2016;11(6).

25. Gibbons HM, Owen R, Heller T. Perceptions of health and healthcare of people with intellectual and developmental disabilities in Medicaid managed care. Intellectual and Developmental Disabilities. 2016;54(2):94-105.

26. Gormez A, Kurtulmus A, Ince Z, Torun P, Uysal O, Citak S. Psychiatric symptoms, challenging behaviour and utilization of psychiatric services among adults with intellectual disabilities in Turkey. Journal of Applied Research in Intellectual Disabilities. 2020;33(5):1038-48.

27. Green AR, Wolff JL, Echavarria DM, Chapman M, Phung A, Smith D, et al. How clinicians discuss medications during primary care encounters among older adults with cognitive impairment. Journal of General Internal Medicine. 2020;35(1):237-46.

28. Guerin BM, Payne DA, Roy DE, McPherson KM. "It's just so bloody hard": Recommendations for improving health interventions and maternity support services for disabled women. Disability and Rehabilitation. 2017;39(23):2395-403.

29. Guzder J, Yohannes S, Zelkowitz P. Helpseeking of immigrant and native born parents: A qualitative study from a Montreal child day hospital. Journal of the Canadian Academy of Child and Adolescent Psychiatry (Journal de l'Academie canadienne de psychiatrie de l'enfant et de l'adolescent). 2013;22(4):275-81.

30. Harris MG, Baxter AJ, Reavley N, Diminic S, Pirkis J, Whiteford HA. Gender-related patterns and determinants of recent help-seeking for past-year affective, anxiety and substance use disorders: Findings from a national epidemiological survey. Epidemiology and Psychiatric Sciences. 2016;25(6):548-61.

31. Hasan AA, Musleh M. Barriers to seeking early psychiatric treatment amongst first-episode psychosis patients: A qualitative study. Issues in Mental Health Nursing. 2017;38(8):669-77.

32. Henderson J, Battams S. Mental health and barriers to the achievement of the 'right to health'. Australian Journal of Primary Health. 2011;17(3):220-6.

33. Hill C, Deville C, Alcorn S, Kiess A, Viswanathan A, Page B. Assessing and providing culturally competent care in radiation oncology for deaf cancer patients. Advances in Radiation Oncology. 2020;5(3):333-44.

34. Horner-Johnson W, Klein KA, Campbell J, Guise J-M. Experiences of women with disabilities in accessing and receiving contraceptive care. Journal of Obstetric, Gynecologic & Neonatal Nursing. 2021;50(6):732-41.

35. Igwesi-Chidobe C. Obstacles to obtaining optimal physiotherapy services in a rural community in southeastern Nigeria. Rehabilitation Research and Practice. 2012;2012.

36. Jindal P, MacDermid JC, Rosenbaum P, DiRezze B, Narayan A. Perspectives on rehabilitation of children with cerebral palsy: Exploring a cross-cultural view of parents from India and Canada using the international classification of functioning, disability and health. Disability and Rehabilitation. 2018;40(23):2745-55.

37. Kaufman EA, McDonell MG, Cristofalo MA, Ries RK. Exploring barriers to primary care for patients with severe mental illness: Frontline patient and provider accounts. Issues in Mental Health Nursing. 2012;33(3):172-80.

38. Kenya Union of the Blind, World Blind Union. Documenting Kenya’s implementation of Sustainable Development Goals (SDGs) in compliance with the UN Convention on the Rights of Persons with Disabilities (CRPD). 2020.

39. Kumurenzi A, Goliath C, Mji G, Mlenzana N, Joseph C, Stathum S, et al. Experiences of patients and service providers with out-patient rehabilitation services in a rehabilitation centre in the Western Cape Province. African Journal of Disability. 2015;4(1).

40. Lee S, Waters F, Briffa K, Fary RE. Limited interface between physiotherapy primary care and people with severe mental illness: A qualitative study. Journal of Physiotherapy. 2017;63(3):168-74.

41. Lewis N, Lewis K, Davies B. ‘I don't feel trapped anymore...I feel like a bird’: People with learning disabilities' experience of psychological therapy. Journal of Applied Research in Intellectual Disabilities. 2016;29(5):445-54.

42. Lindsay S, King G, Klassen AF, Esses V, Stachel M. Working with immigrant families raising a child with a disability: Challenges and recommendations for healthcare and community service providers. Disability and Rehabilitation. 2012;34(23):2007-17.

43. Makhoba M, Joseph N. Practices and views of audiologists regarding aural rehabilitation services for adults with acquired hearing loss. The South African Journal of Communication Disorders. 2016;63(1):e1-e10.

44. Man J, Kangas M. Service satisfaction and helpfulness ratings, mental health literacy and help seeking barriers of carers of individuals with dual disabilities. Journal of Applied Research in Intellectual Disabilities. 2019;32(1):184-93.

45. Marthoenis M, Aichberger MC, Schouler-Ocak M. Patterns and determinants of treatment seeking among previously untreated psychotic patients in Aceh Province, Indonesia: A qualitative study. Scientifica. 2016;2016.

46. McBain H, Mulligan K, Lamontagne-Godwin F, Jones J, Haddad M, Flood C, et al. Implementation of recommended type 2 diabetes care for people with severe mental illness: A qualitative exploration with healthcare professionals. BMC Psychiatry. 2016;16.

47. Mimmo L, Woolfenden S, Travaglia J, Harrison R. Partnerships for safe care: A meta-narrative of the experience for the parent of a child with intellectual disability in hospital. Health Expectations. 2019;22(6):1199-212.

48. Mitchell R, Fajardo Pulido D, Ryder T, Norton G, Brodaty H, Draper B, et al. Access to rehabilitation services for older adults living with dementia or in a residential aged care facility following a hip fracture: Healthcare professionals' views. Disability and Rehabilitation. 2021;43(6):834-45.

49. Mitra M, Akobirshoev I, Moring NS, Long-Bellil L, Smeltzer SC, Smith LD, et al. Access to and satisfaction with prenatal care among pregnant women with physical disabilities: Findings from a national survey. Journal of Women’s Health. 2017;26(12):1356-63.

50. Mkabile S, Swartz L. Putting cultural difference in its place: Barriers to access to health services for parents of children with intellectual disability in an urban African setting. International Journal of Social Psychiatry. 2021;68(8):1614-22.

51. Morris MA, Yorkston K, Clayman ML. Improving communication in the primary care setting: Perspectives of patients with speech disabilities. The Patient - Patient-Centered Outcomes Research. 2014;7(4):397-401.

52. Murphy KA, Stone EM, Presskreischer R, McGinty EE, Daumit GL, Pollack CE. Cancer screening among adults with and without serious mental illness: A mixed methods study. Medical Care. 2021;59(4):327-33.

53. National Council on Disability. Medical futility and disability bias: Part of the bioethics and disability series. Washington, DC; 2019.

54. National Council on D. Enforceable Accessible Medical Equipment Standards: A Necessary Means to Address the Health Care Needs of People with Mobility Disabilities. Washington, CD; 2021.

55. Nguyen A, Liamputtong P, Horey D. Reproductive health care experiences of people with physical disabilities in Vietnam. Sexuality and Disability. 2019;37(3):383-400.

56. Novak P, Feder KA, Ali MM, Chen J. Behavioral health treatment utilization among individuals with co-occurring opioid use disorder and mental illness: Evidence from a national survey. Journal of Substance Abuse Treatment. 2019;98:47-52.

57. Nuri RP, Aldersey HM, Ghahari S, Huque AS. Service providers' perspectives in providing services to children with disabilities and their families in Bangladesh. Disability and Rehabilitation. 2022;44(17):4700-8.

58. O'Halloran R, Grohn B, Worrall L. Environmental factors that influence communication for patients with a communication disability in acute hospital stroke units: A qualitative metasynthesis. Archives of Physical Medicine and Rehabilitation. 2012;93(1):S77-85.

59. Oyelade OO, Nkosi-Mafutha NG. Living beyond the limitation: Rehabilitation, life and productivity of individuals with schizophrenia in South-West Nigeria. Health Expectations. 2021;24(2):198-208.

60. Pearson J, Payne D, Yoshida K, Garrett N. Access to and engagement with cervical and breast screening services for women with disabilities in Aotearoa New Zealand. Disability and Rehabilitation. 2022;44(10):1984-95.

61. Peters K, Cotton A. Barriers to breast cancer screening in Australia: Experiences of women with physical disabilities. Journal of Clinical Nursing. 2015;24(3-4):563-72.

62. Pitman AL, Osborn DPJ, Wright CA, Nazareth I, King MB. Cardiovascular screening of people with severe mental illness in England: Views of service users and providers. Psychiatric Services. 2011;62(11):1338-45.

63. Poon WC, Joubert L, Harvey C. Experiences of Chinese migrants caring for family members with schizophrenia in Australia. Social Work in Health Care. 2013;52(2-3):144-65.

64. Porat O, Heruti R, Navon-Porat H, Hardoff D. Counseling young people with physical disabilities regarding relationships and sexuality issues: Utilization of a novel service. Sexuality and Disability. 2012;30(3):311-7.

65. Redfern H, Burton J, Lonne B, Seiffert H. Social work and complex care systems: The case of people hospitalised with a disability. Australian Social Work. 2016;69(1):27-38.

66. Relyea E, MacDonald B, Cattaruzza C, Marshall D. On the margins of death: A scoping review on palliative care and schizophrenia. Journal of Palliative Care. 2019;34(1):62-9.

67. Roux-Levy P-H, Sanlaville D, De Freminville B, Touraine R, Masurel A, Gueneau I, et al. Care management in a French cohort with Down Syndrome from the AnDDI-Rares/CNSA study. European Journal of Medical Genetics. 2021;64(10):104290-.

68. Sakellariou D, Rotarou ES. Access to healthcare for men and women with disabilities in the UK: Secondary analysis of cross-sectional data. BMJ Open. 2017;7(8):e016614-e.

69. Saleeby PW, Hunter-Jones J. Identifying barriers and facilitators to breast health services among women with disabilities. Social Work in Public Health. 2016;31(4):255-63.

70. Samtani G, Bassford TL, Williamson HJ, Armin JS. Are researchers addressing cancer treatment and survivorship among people with intellectual and developmental disabilities in the US? A scoping review. Intellectual and Developmental Disabilities. 2021;59(2):141-54.

71. Schildberger B, Zenzmaier C, Konig-Bachmann M. Experiences of Austrian mothers with mobility or sensory impairments during pregnancy, childbirth and the puerperium: A qualitative study. BMC Pregnancy and Childbirth. 2017;17(1).

72. Sightsavers. Knowledge, attitudes and practices in eye health and disability in Sierra Leone: Study Report 2014. 2014 2014.

73. Silva VAd, Busnello ARR, Cavassin RC, Loureiro APC, Moser ADdL, Carvalho DR. Physiotherapy access for children and adolescents with physical disabilities in public institutions. Ciência & Saude Coletiva. 2020;25(7):2859-70.

74. Smeltzer SC, Mitra M, Long-Bellil L, Iezzoni LI, Smith LD. Obstetric clinicians' experiences and educational preparation for caring for pregnant women with physical disabilities: A qualitative study. Disability and Health Journal. 2018;11(1):8-13.

75. Tabril T, Chekira A, Touhami YOH, El Allani L, Najid I, Hammani Z, et al. The role of the general practitioner in management of psychiatric disorders. Revue d’Épidemiologie et de Santé Publique. 2020;68(3):185-92.

76. Tarasoff LA. "We don't know. We've never had anybody like you before": Barriers to perinatal care for women with physical disabilities. Disability and Health Journal. 2017;10(3):426-33.

77. Tuffrey-Wijne I, Goulding L, Giatras N, Abraham E, Gillard S, White S, et al. The barriers to and enablers of providing reasonably adjusted health services to people with intellectual disabilities in acute hospitals: Evidence from a mixed-methods study. BMJ Open. 2014;4(4):1-10.

78. UNICEF. Best of UNICEF research 2017. Innocenti, Florence: UNICEF Office of Research; 2017.

79. Voillemont C, Imbault E, Schoenberger M, Di Patrizio P. Care and management of adults with autism spectrum disorder in family practice: Difficulties experienced by general practitioners. Family Practice. 2022;39(3):464-70.

80. Webb CM, Collin SM, Deave T, Haig-Ferguson A, Spatz A, Crawley E. What stops children with a chronic illness accessing health care: A mixed methods study in children with Chronic Fatigue Syndrome/Myalgic Encephalomyelitis (CFS/ME). BMC Health Services Research. 2011;11.

81. Wheeler AC, Wylie A, Villagomez A, Bishop E, Raspa M. Health care for individuals with fragile X Syndrome: Understanding access and quality. Disability and Health Journal. 2019;12(2):269-77.

82. Wong JL, Alschuler KN, Mroz TM, Hreha KP, Molton IR. Identification of targets for improving access to care in persons with long term physical disabilities. Disability and Health Journal. 2019;12(3):366-74.

83. Zuurmond M, Mactaggart I, Kannuri N, Murthy G, Oye JE, Polack S. Barriers and facilitators to accessing health services: A qualitative study amongst people with disabilities in Cameroon and India. International Journal of Environmental Research and Public Health. 2019;16(7).

84. World Health Organization. WHO regional offices 2023 [Available from: <https://www.who.int/about/who-we-are/regional-offices>.

85. World Bank. The World Bank’s country classification by income 2023 [Available from: <https://datatopics.worldbank.org/world-development-indicators/the-world-by-income-and-region.html>.

86. UNDESA. UNDESA’s description of the Convention on the Rights of Persons with Disabilities (CRPD). 2023.

87. Kelly C, Dansereau L, Sebring J, Aubrecht K, FitzGerald M, Lee Y, et al. Intersectionality, health equity, and EDI: What's the difference for health researchers? International Journal for Equity in Health. 2022;21(1).

88. United Nations. Vulnerable groups: who are they? 2023 [3 October 2023]. Available from: <https://www.un.org/en/fight-racism/vulnerable-groups>.

89. United Nations. Convention on the Rights of Persons with Disabilities (UNCRPD). New York; 2006.

90. Alduhaim A, Purcell A, Cumming S, Doble M. A new training package (3Cs: Connect, Communicate and Collaborate) for improving family responsive service delivery in early intervention for children with hearing loss: A proof of concept study. International Journal of Pediatric Otorhinolaryngology. 2021;140:110484-.

91. Amin R, Thomas MA. Effects of brief depression and anxiety management training on a US army division's primary care providers. Military Medicine. 2020;185(5):e719-e23.

92. Archibald D, Stratton J, Liddy C, Grant RE, Green D, Keely EJ. Evaluation of an electronic consultation service in psychiatry for primary care providers. BMC Psychiatry. 2018;18(1).

93. Armstrong M, Morris C, Abraham C, Tarrant M. Interventions utilising contact with people with disabilities to improve children's attitudes towards disability: A systematic review and meta-analysis. Disability and Health Journal. 2016;10(1):11-22.

94. Aviram U, Ginath Y, Roe D. Mental health reforms in Europe: Israel's rehabilitation in the community of persons with mental disabilities law: Challenges and opportunities. Psychiatric Services. 2012;63(2):110-2.

95. Barr M, Duncan J, Dally K. Parent experience of the national disability insurance scheme (NDIS) for children with hearing loss in Australia. Disability & Society. 2021;36(10):1663-87.

96. Battistella LR, Juca SSH, Tateishi M, Oshiro MS, Yamanaka EI, Lima E, et al. Lucy Montoro Rehabilitation Network mobile unit: An alternative public healthcare policy. Disability and Rehabilitation: Assistive Technology. 2015;10(4):309-15.

97. Breslau J, Leckman-Westin E, Han B, Guarasi D, Yu H, Horvitz-Lennon M, et al. Providing health physicals and/or health monitoring services in mental health clinics: Impact on laboratory screening and monitoring for high risk populations. Administration and Policy in Mental Health and Mental Health Services Research. 2021;48(2):279-89.

98. Broussard B, Radkins JB, Compton MT. Developing visually based, low-literacy health education tools for African Americans with psychotic disorders and their Families. Community Mental Health Journal. 2014;50(6):629-36.

99. Brown CM, Beck AF, Steuerwald W, Alex, er E, Samaan ZM, et al. Narrowing care gaps for early language delay: A quality improvement study. Clinical Pediatrics. 2016;55(2):137-44.

100. Buchan CA. Therapeutic benefits and limitations of participatory photography for adults with mental health problems: A systematic search and literature review. Journal of Psychiatric and Mental Health Nursing. 2020;27(5):657-68.

101. Buszewicz M, Welch C, Horsfall L, Nazareth I, Osborn D, Hassiotis A, et al. Assessment of an incentivised scheme to provide annual health checks in primary care for adults with intellectual disability: A longitudinal cohort study. Lancet Psychiatry. 2014;1(7):522-30.

102. Byrne L, Happell B, Welch T, Moxham LJ. “Things you can't learn from books”: Teaching recovery from a lived experience perspective. International Journal of Mental Health Nursing. 2013;22(3):195-204.

103. Callanan J, Signal T, McAdie T. What is my child telling me? Reducing stress, increasing competence and improving psychological well-being in parents of children with a developmental disability. Research in Developmental Disabilities. 2021;114.

104. Chien WT, Leung SF, Chu CSK. A nurse-led, needs-based psycho-education intervention for Chinese patients with first-onset mental illness. Contemporary Nurse. 2012;40(2):194-209.

105. Collins RL, Wong EC, Breslau J, Burnam MA, Cefalu M, Roth E. Social marketing of mental health treatment: California's mental illness stigma reduction campaign. American Journal of Public Health. 2019;109:S228-S35.

106. Cook JA, Jonikas JA, Hamilton MM, Goldrick V, Steigman PJ, Grey DD, et al. Impact of Wellness Recovery Action Planning on service utilization and need in a randomized controlled trial. Psychiatric Rehabilitation Journal. 2013;36(4):250-7.

107. Corrigan P, Sheehan L, Morris S, Larson JE, Torres A, Lara JL, et al. The impact of a peer navigator program in addressing the health needs of Latinos with serious mental illness. Psychiatric Services. 2018;69(4):456-61.

108. Crowley R, Wolfe I, Lock K, McKee M. Improving the transition between paediatric and adult healthcare: A systematic review. Archives of Disease in Childhood. 2011;96(6):548-53.

109. D’Aprano A, Gibb S, Riess S, Cooper M, Mountford N, Meehan E. Important components of a programme for children with medical complexity: An Australian perspective. Child: Care, Health and Development. 2020;46(1):90-103.

110. Dagnan D, Masson J, Thwaites R, James A, Hatton C. Training therapists to work with people with intellectual disability in Improving Access to Psychological Therapies (IAPT) services. Journal of Applied Research in Intellectual Disabilities. 2018;31(5):760-7.

111. Devine A, Ignacio R, Prenter K, Temminghoff L, Gill-Atkinson L, Zayas J, et al. "Freedom to go where I want": Improving access to sexual and reproductive health for women with disabilities in the Philippines. Reproductive Health Matters. 2017;25(50):55-65.

112. du Toit R, Courtright P, Lewallen S. The use of key informant method for identifying children with blindness and severe visual impairment in developing countries. Ophthalmic Epidemiology. 2017;24(3):153-67.

113. Fiander AN, Vanneste T. transportMYpatient: An initiative to overcome the barrier of transport costs for patients accessing treatment for obstetric fistulae and cleft lip in Tanzania. Tropical Doctor. 2012;42(2):77-9.

114. Evans-Lacko S, Henderson C, Thornicroft G, McCrone P. Economic evaluation of the anti-stigma social marketing campaign in England 2009-2011. British Journal of Psychiatry. 2013;202(s55):s95-s101.

115. Feldman MA, Owen F, Andrews A, Hamelin J, Barber R, Griffiths D. Health self-advocacy training for persons with intellectual disabilities: Health self-advocacy training. Journal of Intellectual Disability Research. 2012;56(11):1110-21.

116. Giesbrecht EM, Miller WC, Mitchell IM, Woodgate RL. Development of a wheelchair skills home program for older adults using a participatory action design approach. BioMed Research International. 2014;2014:172434-.

117. Gillies D, Buykx P, Parker AG, Hetrick SE. Consultation liaison in primary care for people with mental disorders. Cochrane Database of Systematic Reviews. 2015(9).

118. Golyk V, Syvak O, Grabljevec K, Tederko P, Gutenbrunner C, Nugraha B. Five years after development of the national disability, health and rehabilitation plan for Ukraine: Achievements and challenges. Journal of Rehabilitation Medicine. 2021;53(3).

119. Grady B, Singleton M. Telepsychiatry “coverage" to a rural Iinpatient psychiatric unit. Telemedicine and e-Health. 2011;17(8):603-8.

120. Gureje O, Appiah-Poku J, Bello T, Kola L, Araya R, Chisholm D, et al. Effect of collaborative care between traditional and faith healers and primary health-care workers on psychosis outcomes in Nigeria and Ghana (COSIMPO): A cluster randomised controlled trial. Lancet. 2020;396(10251):612-22.

121. Hamblen JL, Grubaugh AL, Davidson TM, Borkman AL, Bunnell BE, Ruggiero KJ. An online peer educational campaign to reduce stigma and improve help seeking in veterans with posttraumatic stress disorder. Telemedicine and e-Health 2019;25(1):41-7.

122. Hamdani Y, Proulx M, Kingsnorth S, Lindsay S, Maxwell J, Colantonio A, et al. The LIFEspan model of transitional rehabilitative care for youth with disabilities: Healthcare professionals' perspectives on service delivery. Journal of Pediatric Rehabilitation Medicine. 2014;7(1):79-91.

123. Hammarberg K, Sartore G, Cann W, Fisher JRW. Barriers and promoters of participation in facilitated peer support groups for carers of children with special needs. Scandinavian Journal of Caring Sciences. 2014;28(4):775-83.

124. Happell B, Byrne L, McAllister M, Lampshire D, Roper C, Gaskin CJ, et al. Consumer involvement in the tertiary-level education of mental health professionals: A systematic review. International Journal of Mental Health Nursing. 2014;23(1):3-16.

125. Harney BL, Brereton R, Whitton B, Pietrzak D, Paige E, Roberts SK, et al. Hepatitis C treatment in a co-located mental health and alcohol and drug service using a nurse-led model of care. Journal of Viral Hepatitis. 2021;28(5):771-8.

126. Henderson C, Noblett J, Parke H, Clement S, Caffrey A, Gale-Grant O, et al. Mental health-related stigma in health care and mental health-care settings. Lancet Psychiatry. 2014;1(6):467-82.

127. Hensley MA. Case managers and the use of Medicare: Part D. Professional Case Management. 2011;16(6):301-8.

128. Highland J, Nikolajski C, Kogan J, Ji Y, Kukla M, Schuster J. Impact of Behavioral Health Homes on cost and utilization outcomes. Psychiatric Services. 2020;71(8):796-802.

129. Irwin KE, Park ER, Fields LE, Corveleyn AE, Greer JA, Perez GK, et al. Bridge: Person-centered collaborative care for patients with serious mental illness and cancer. The Oncologist. 2019;24(7):901-10.

130. Isaacs AN, Sutton K, Dalziel K, Maybery D. Outcomes of a care coordinated service model for persons with severe and persistent mental illness: A qualitative study. International Journal of Social Psychiatry. 2017;63(1):40-7.

131. Johnson K, Tepper M, Leff HS, Mullin BO, Cook BL, Progovac AM. Assessing the long-term effectiveness of a Behavioral Health Home for adults with bipolar and psychotic disorders. Psychiatric Services. 2021;73(2):172-9.

132. Jokel R, Meltzer J, D R J, D M L, J C J, A N E, et al. Group intervention for individuals with primary progressive aphasia and their spouses: Who comes first? Journal of Communication Disorders. 2017;66:51-64.

133. Jones M, Kruger M, Walsh SM. Preparing non-government organization workers to conduct health checks for people with serious mental illness in regional Australia. Journal of Psychiatric and Mental Health Nursing. 2016;23(5):247-54.

134. Kennedy J, Wood EG, Frieden L. Disparities in insurance coverage, health services use, and access following implementation of the Affordable Care Act: A comparison of disabled and nondisabled working-age adults. Inquiry Journal. 2017;54.

135. Kilbourne AM, Goodrich DE, Lai Z, Almirall D, Nord KM, Bowersox NW, et al. Reengaging veterans with serious mental illness into care: Preliminary results from a national randomized trial. Psychiatric Services. 2015;66(1):90-3.

136. King JA, King MJ, Edwards N, Hair SA, Cheang S, Pearson A, et al. Addressing transport safety and accessibility for people with a disability in developing countries: A formative evaluation of the Journey Access Tool in Cambodia. Global Health Action. 2018;11(1).

137. Kranz AM, Ross R, Sorbero M, Kofner A, Stein BD, Dick AW. Impact of a Medicaid policy on preventive oral health services for children with intellectual disabilities, developmental disabilities, or both. Journal of the American Dental Association. 2020;151(4):255-64.

138. Kreutzberg A, Jacobs R. Improving access to services for psychotic patients: Does implementing a waiting time target make a difference. European Journal of Health Economics. 2020;21(5):703-16.

139. Lam M, Li LH, Anderson KK, Shariff SZ, Forchuk C. Evaluation of the transitional discharge model on use of psychiatric health services: An interrupted time series analysis. Journal of Psychiatric and Mental Health Nursing. 2020;27(2):172-84.

140. Li J, Fan Y, Zhong HQ, Duan XL, Chen W, Evans-Lacko S, et al. Effectiveness of an anti-stigma training on improving attitudes and decreasing discrimination towards people with mental disorders among care assistant workers in Guangzhou, China. International Journal of Mental Health Systems. 2019;13(1).

141. Lovero KL, Lammie SL, van Zyl A, Paul SN, Ngwepe P, Mootz JJ, et al. Mixed-methods evaluation of mental healthcare integration into tuberculosis and maternal-child healthcare services of four South African districts. BMC Health Services Research. 2019;19(1).

142. Macdonald S, Morrison J, Melville CA, Baltzer M, MacArthur L, Cooper SA. Embedding routine health checks for adults with intellectual disabilities in primary care: Practice nurse perceptions. Journal of Intellectual Disability Research. 2018;62(4):349-57.

143. Mackinnon J, Murphy H. "I used to think that they were all abnormal. And I was the normal one": Conceptualizing mental health and mental health treatment under Improving Access to Psychological Therapies (IAPT). Journal of Mental Health. 2016;25(5):428-33.

144. Malik NN, Kannusamy P, Klanin-Yobas P. The effectiveness of mental health-related theoretical education and clinical placement in mental health settings in changing the attitudes of health care students towards mental illness: A systematic review. Joanna Briggs Institute Library of Systematic Reviews. 2012;10(58):4019-76.

145. Martin-Prudent A, Lartz M, Borders C, Meehan T. Early intervention practices for children with hearing loss: Impact of professional development. Communication Disorders Quarterly. 2016;38(1):13-23.

146. Mathias K, Mathias J, Goicolea I, Kermode M. Strengthening community mental health competence-A realist informed case study from Dehradun, North India. Health & Social Care in the Community. 2018;26(1):e179-e90.

147. McClellan C, Maclean JC, Saloner B, McGinty EE, Pesko MF. Integrated care models and behavioral health care utilization: Quasi-experimental evidence from Medicaid health homes. Health Economics. 2020;29(9):1086-97.

148. Mehta N, Clement S, Marcus E, Stona AC, Bezborodovs N, Evans-Lacko S, et al. Evidence for effective interventions to reduce mental health-related stigma and discrimination in the medium and long term: Systematic review. British Journal of Psychiatry. 2015;207(5):377-84.

149. Mejia-Lancheros C, Lachaud J, To MJ, Lee P, Nisenbaum R, O'Campo P, et al. The long-term effects of a Housing First intervention on primary care and non-primary care physician visits among homeless adults with mental illness: A 7-Year RCT follow-up. Journal of Primary Care & Community Health. 2021;12.

150. Mittal D, Sullivan G, Chekuri L, Allee E, Corrigan PW. Empirical studies of self-stigma reduction strategies: A critical review of the literature. Psychiatric Services. 2012;63(10):974-81.

151. Morabito MS, Savage J, Sneider L, Wallace K. Police response to people with mental illnesses in a major U.S. city: The Boston experience with the co-responder model. Victims & Offenders. 2018;13(8):1093-105.

152. Morriss R, Vinjamuri I, Faizal MA, Bolton CA, McCarthy JP. Training to recognise the early signs of recurrence in schizophrenia. Cochrane Database of Systematic Reviews. 2013;28(2).

153. Moxham L, Patterson C, Taylor E, Perlman D, Sumskis S, Brighton R. A multidisciplinary learning experience contributing to mental health rehabilitation. Disability and Rehabilitation. 2017;39(1):98-103.

154. Mudrick NR, Breslin ML, Nielsen KA, Swager LC. Can disability accommodation needs stored in electronic health records help providers prepare for patient visits? A qualitative study. BMC Health Services Research. 2020;20(1).

155. Mueller-Stierlin AS, ra, Helmbrecht MJ, Herder K, Prinz S, Rosenfeld N, et al. Does one size really fit all? The effectiveness of a non-diagnosis-specific integrated mental health care program in Germany in a prospective, parallel-group controlled multi-centre trial. BMC Psychiatry. 2017;17(1).

156. Murphy KA, Daumit GL, Stone E, McGinty EE. Physical health outcomes and implementation of behavioural health homes: A comprehensive review. International Review of Psychiatry. 2018;30(6):224-41.

157. Musyimi CW, Mutiso VN, Nandoya ES, Ndetei DM. Forming a joint dialogue among faith healers, traditional healers and formal health workers in mental health in a Kenyan setting: Towards common grounds. Journal of Ethnobiology and Ethnomedicine. 2016;12(4).

158. Neherta M, Maisa E, Sari Y. Intervention of sexual abuse prevention for mother of children with mental retardation in Payakumbuh, Indonesia, 2016. Indian Journal of Public Health Research & Development. 2019;10(1):461-6.

159. O'Donovan J, Namanda AS, Hamala R, Winters N, Bhutta MF. Exploring perceptions, barriers, and enablers for delivery of primary ear and hearing care by community health workers: A photovoice study in Mukono District, Uganda. International Journal for Equity in Health. 2020;19(1).

160. Oliveira GOB, Cavalcante LDW, Pagliuca LMF, Almeida PCd, Rebouças CBdA. Prevention of sexually transmitted diseases among visually impaired people: Educational text validation. Revista Latino-Americana de Enfermagem. 2016;24.

161. Oliveira MGd, Áfio ACE, Almeida PCd, Machado MMT, Lindsay AC, Pagliuca LMF. Teaching blind women about the anatomy and physiology of the female reproductive system through educational manual. Revista Brasileira de Saúde Materno Infantil. 2018;18(4):755-61.

162. Owen R, Crabb C, Stober K, Mitchell D, Yamaki K, Heller T. Utilization of and relationships with primary care providers during the transition to Medicaid managed care. Journal of Disability Policy Studies. 2020;31(2):67-76.

163. Parish SL, Rose RA, Yoo J, Swaine JG. State Medicaid policies and the health care access of low-income children with special health care needs living in the American South. North Carolina Medical Journal. 2012;73(1):15-23.

164. Pathare S, Funk M, Drew Bold N, Chauhan A, Kalha J, Krishnamoorthy S, et al. Systematic evaluation of the QualityRights programme in public mental health facilities in Gujarat, India. British Journal of Psychiatry. 2021;218(4):196-203.

165. Pennybaker S, Hemming P, Roy D, Anton B, Chisolm MS. Risks, benefits, and recommendations for pastoral care on inpatient psychiatric units: A systematic review. Journal of Psychiatric Practice. 2016;22(5):363-81.

166. Poltorak M. Anthropology, brokerage, and collaboration in the development of a Tongan public psychiatry: Local lessons for global mental health. Transcultural Psychiatry. 2016;53(6):743-65.

167. Porter E, Kidd G, Murray N, Uytman C, Spink A, Anderson B. Developing the pregnancy support pack for people who have a learning disability. British Journal of Learning Disabilities. 2012;40(4):310-7.

168. Rai S, Gurung D, Kaiser BN, Sikkema KJ, Dhakal M, Bhardwaj A, et al. A service user co-facilitated intervention to reduce mental illness stigma among primary healthcare workers: Utilizing perspectives of family members and caregivers. Families Systems & Health. 2018;36(2):198-209.

169. Rico-Blázquez M, García-Sanz P, Martín-Martín M, Lopez-Rodríguez JA, Morey-Montalvo M, Sanz-Cuesta T, et al. Effectiveness of a home-based nursing support and cognitive restructuring intervention on the quality of life of family caregivers in primary care: A pragmatic cluster-randomized controlled trial. International Journal of Nursing Studies. 2021;120.

170. Ride J, Kasteridis P, Gutacker N, Kronenberg C, Doran T, Mason A, et al. Do care plans and annual reviews of physical health influence unplanned hospital utilisation for people with serious mental illness? Analysis of linked longitudinal primary and secondary healthcare records in England. BMJ Open. 2018;8(11).

171. Robles-Bykbaev Y, Oyola-Flores C, Robles-Bykbaev VE, López-Nores M, Ingavélez-Guerra P, Pazos-Arias JJ, et al. A bespoke social network for deaf women in Ecuador to access information on sexual and reproductive health. International Journal of Environmental Research and Public Health. 2019;16(20).

172. Röhricht F, Padmanabhan R, Binfield P, Mavji D, Barlow S. Simple mobile technology health management tool for people with severe mental illness: A randomised controlled feasibility trial. BMC Psychiatry. 2021;21(1).

173. Romaire M, Alterbaum R, Collins A. Medicaid Behavioral Health Homes: Lessons learned and early findings from Maine. Psychiatric Services. 2020;71(11):1179-87.

174. Rotenberg S, Rodríguez Gatta D, Wahedi A, Loo R, McFadden E, Ryan S. Disability training for health workers: A global evidence synthesis. Disability and Health Journal. 2022;15(2):101260-.

175. Schilling S, Bustamante JA, Sala A, Acevedo C, Tapia E, Alvarado R, et al. Development of an intervention to reduce self-stigma in outpatient mental health service users in Chile. Revista de la Facultad de Ciencias Medicas. 2015;72(4):284-94.

176. Shor R, Shalev A. The significance of services in a psychiatric hospital for family members of persons with mental illness. Families Systems & Health. 2015;33(1):68-71.

177. Smythe T, Adelson JD, Polack S. Systematic review of interventions for reducing stigma experienced by children with disabilities and their families in low- and middle-income countries: State of the evidence. Tropical Medicine & International Health. 2020;25(5):508-24.

178. Steinert C, Steinert T, Flammer E, Jaeger S. Impact of the UN convention on the rights of persons with disabilities (UN-CRPD) on mental health care research: A systematic review. BMC Psychiatry. 2016;16.

179. Steele C, Ungemack J, Mormile-Mehler M, Rabitaille W. Changes in hospital utilization among seriously mentally ill patients following enrollment in aniIntegrated primary and behavioral health care program. Connecticut Medicine. 2017;81(5):271-9.

180. Thornicroft GP, Mehta NM, Clement SP, Evans-Lacko SP, Doherty MM, Rose DP, et al. Evidence for effective interventions to reduce mental-health-related stigma and discrimination. Lancet. 2015;387(10023):1123-32.

181. Tran N, Castle D. Outcomes from a regular medication information programme for consumers with a mental illness. Australasian Psychiatry. 2012;20(2):143-7.

182. Trondsen MV, Tjora A, Broom A, Scambler G. The symbolic affordances of a video-mediated gaze in emergency psychiatry. Social Science & Medicine. 2018;197:87-94.

183. Vaughan C, Gill-Atkinson L, Devine A, Zayas J, Ignacio R, Garcia J, et al. Enabling action: Reflections upon inclusive participatory research on health with women with disabilities in the Philippines. American Journal of Community Psychology. 2020;66(3):370-80.

184. Velligan DI, Roberts D, Martinez M, Fredrick M, Hillner K, Luber P. Following AACP guidelines for transitions in care: The transitional care clinic. Psychiatric Services. 2016;67(3):259-61.

185. Willems A, Iyamuremye JD, Misage CN, Smith-Swintosky V, Kayiteshonga Y. Co-creation and Evaluation of Nationwide Remote Training Service for Mental Health Education of Community Health Workers in Rwanda. Frontiers in Public Health. 2021;9.

186. Williams A, Fossey E, Farhall J, Foley F, Thomas N. Impact of jointly using an e-mental health resource (self-management and recovery technology) on interactions between service users experiencing severe mental illness and community mental health workers: Grounded theory study. Journal of Medical Internet Research. 2021;8(6).

187. Xiong GL, Iosif A-M, Suo S, McCarron RM, Koike A, Onate J, et al. Understanding preventive health screening services use in persons with serious mental illness: How does integrated behavioral health primary care compare? International Journal of Psychiatry in Medicine. 2015;48(4):279-98.

188. Yanos PT, Lucksted A, Drapalski AL, Roe D, Lysaker P. Interventions targeting mental health self-stigma: A review and comparison. Psychiatric Rehabilitation Journal. 2015;38(2):171-8.
